# Nutritional Composition of Forage Available to the Northern Hairy‐Nosed Wombat

**DOI:** 10.1002/ece3.70514

**Published:** 2024-11-07

**Authors:** Fiona F. Casey, Julie M. Old, Hayley J. Stannard

**Affiliations:** ^1^ School of Science Western Sydney University Hawkesbury New South Wales Australia; ^2^ School of Agricultural, Environmental and Veterinary Sciences Charles Sturt University Wagga Wagga New South Wales Australia

**Keywords:** buffel grass, conservation, critically endangered, endangered species, *Lasiorhinus krefftii*, nutrition

## Abstract

Access to adequate nutrition supports an animal's chance of survival and reproduction; thus, it is particularly important for threatened species. The nutritional quality of forage available to the critically endangered northern hairy‐nosed wombat (*Lasiorhinus krefftii*; NHW) has not been assessed for two decades. The NHW Recovery Action Plan 2022 highlighted a need to investigate the effects of invasive buffel grass (*Cenchrus ciliaris*) on the species' diet – reassessing the relative nutritional quality of highly abundant buffel grass will assist this investigation. This study assessed the nutritional composition of NHW food items, including buffel grass. Comparisons of the nutritional composition were made between two geographically distanced sites (both eucalyptus woodland and savannah with highly abundant buffel grass), seasons and plant genera. The nitrogen, gross energy, acid detergent fibre, neutral detergent fibre, ash and mineral content of plants and their relationship to scats, as faecal nitrogen is a good predictor of palatability of grass and nutritional status, were assessed. The nutritional content of plants varied significantly between sites, seasons and genera. Total forage nutritional quality was greatest during spring 2020 at Epping Forest National Park, during summer 2020/21 and autumn 2021 at Richard Underwood Nature Refuge and poorest during winter at both sites. Buffel grass may be a nutritionally valuable food item of the NHW during winter at both sites. There was no significant relationship between the N and gross energy in forage and scats. The findings of this study will inform management if there is a need to reduce invasive buffel grass, based on enhanced knowledge of the NHW nutritional requirements, by determining whether the species has access to suitable dietary items and meeting their nutritional requirements, particularly when forage quality is poorest or promotion of nutritionally valuable forage items is required. The study will also inform management of nutritional requirements at future translocation sites for the NHW.

## Graphical abstract

The northern hairy‐nosed wombat is a critically endangered species of which there are only about 350 individuals left in the wild, with no captive populations. This article details seasonal and site differences in the nutrient composition of food items in wombat habitats. The data will aid current management of habitats for diet (e.g. increasing preferred grasses in the habitat) and identify habitats with food items of appropriate nutrient compositions for future translocations.

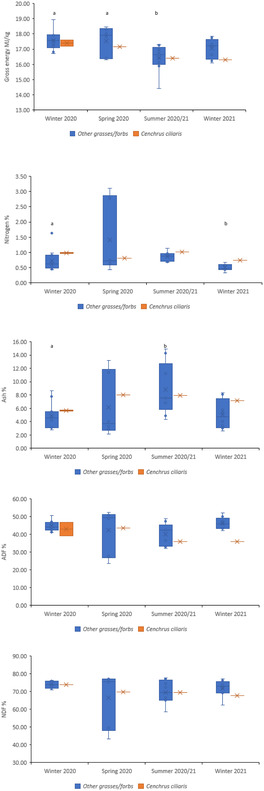

## 1 Introduction

Maintaining biological diversity is essential for providing clean air, water and fertile soils to ensure optimal ecosystem functioning. Australia has a rich biodiversity with approximately 80% of the mammals being endemic (Burbidge et al. 2008); however, these species have undergone significant decline due to habitat destruction, predation, agricultural land practices and other human induced factors. A total of nine mammals are listed as critically endangered (Department of Agriculture Water and the Environment 2024) and that includes one of Australia's largest grazing herbivores: the northern hairy‐nosed wombat (*Lasiorhinus krefftii*; NHW) (Taggart, Martin, and Horsup 2016). Wombats are ecological engineers; they turn over soils through their digging behaviour which in turn contributes to positive changes in nutrient composition of soil and plant growth (Davidson, Detling, and Brown 2012; Guy and Kirkpatrick 2021). In addition, the burrows wombats create provide a refuge for other vertebrates from predators and thermal extremes (Old, Hunter, and Wolfenden 2018; Pike and Mitchell 2013).

There are approximately 315 NHWs across two populations inside predator‐proof fenced areas: one naturally occurring population at Epping Forest National Park (EFNP) in central Queensland (around 300 individuals) and one translocated population at Richard Underwood Nature Refuge (RUNR) in southern Queensland (around 15 individuals) (Department of Environment and Science 2021). The population has grown since the 1980s when the population was estimated at around 35 individuals (Department of Environment and Science 2022). No captive breeding programmes exist for the species, as it is cryptic and skittish, and despite the other two species of extant wombats have been held in captivity successfully, breeding is notoriously difficult and as numbers of the NHW are slowly increasing within the two sites, deemed superfluous (Department of Environment and Science 2022). A primary goal of the recovery plan for the NHW is to establish additional populations throughout the species' former range (Department of Environment and Science 2022). Recently, a new site was identified, based on habitat preference (Department of Environment and Science 2022), to establish a third population of NHWs by translocating individuals from the existing populations (Queensland Cabinet and Ministerial Directory 2023).

Numerous animals have been translocated for the purposes of species conservation (e.g., Armstrong et al. 2015; Clayton et al. 2014; Langford and Burbidge 2001), and availability of high‐quality forage has been linked to a greater rate of success for species translocations in herbivores (Ostermann, Deforge, and Edge 2001; Tarszisz, Dickman, and Munn 2014). Patches of forage with high nutrient densities influence animal body condition and their ability to survive through harsh conditions such as drought or low rainfall (Grant and Scholes 2006). Thus, understanding nutrient availability and intake within an ecosystem is an important component of species conservation. Previously, nutrition has been associated with success of reintroduction programmes for herbivorous ungulates in Asia and Africa. A study on banteng (*Bos javanicus*) showed that nutrition after translocation was higher than before release, contributing to the success of the translocation (Chaiyarat et al. 2020). Similarly, for reintroduced zebra (*Equus burchelli*) and blue wildebeest (*Connochaetes taurinus*), nutritional quality of grasses as well as faecal protein and phosphorus indicated that the environment provided adequate nutrition and animals were well nourished (Mandlate and Rodrigues 2020).

The nutritional composition of wombat dietary items at EFNP have been assessed in the past (Woolnough and Foley 2002). The composition of the vegetation community at RUNR has been described (Department of Environment and Science 2018), but the nutritional composition of forage at RUNR has not been assessed. Due to the critically endangered status of the NHW, experimental research which would typically be conducted in a captive setting, such as determining nutrient requirements, has not been possible; thus, estimates of dietary requirements are derived from those of the southern hairy‐nosed (*Lasiorhinus latifrons*) and bare‐nosed wombats (*Vombatus ursinus*). The hairy‐nosed wombat species are more adapted to living in arid regions compared to the bare‐nosed that occupies more temperate regions. Experiments conducted by Barboza, Hume, and Nolan (1993) showed that both species experienced negative N balance when fed a diet containing 0.6% N, and the estimated N requirement for bare‐nosed wombats was 158 and 201 mg kg^−0.75^ d^−1^ for southern hairy‐nosed wombats. Bare‐nosed and southern hairy‐nosed wombats have relatively low energy requirements (140 kJ kg^−0.75^ d^−1^), much lower than other herbivorous marsupials (Barboza, Hume, and Nolan 1993). Field metabolic rates of all three wombat species of wombat are also lower (132–322 kJ kg d^−1^) than predicted for herbivorous marsupials with significant seasonal differences (Evans, Green, and Newgrain 2003).

In recent decades, the prevalence of invasive buffel grass (*Cenchrus ciliaris*) has increased in biomass dramatically within EFNP (Department of Environment and Science 2022), leading to an increase of buffel grass in the diet of the NHW from ~2% to up to 60% (Casey, Old, and Stannard 2023; Crossman 1988; Flosser 1986; Woolnough. 1998). The occurrence of buffel grass throughout RUNR has also increased (Department of Environment and Science 2018). Recent analysis of the diet of NHW at this site has shown that buffel grass dominates the diet (Casey, Old, and Stannard 2023). The digestibility of buffel grass by the NHW, or any wombat species, has yet to be investigated, and likely differs compared to other species due to differences in digestive anatomy, physiology and requirements of the species. However, when ingested by sheep and cattle, buffel grass has poor digestibility (dry matter digestibility 48%–62%), that varied according to its phenological stage and rainfall, but both species were able to meet their energy requirements for non‐reproductive individuals (Dixon and Coates 2010; Playne 1970).

Buffel grass has been identified as a threat to the vegetation communities at EFNP and RUNR, and a threat to the NHW. Investigating the effects of buffel grass on the diet of the NHW is a species recovery plan goal (Department of Environment and Science 2022). Buffel grass has been of concern for other endangered Australian mammals, such as the bridled nail‐tail wallaby (*Onychogalea fraenata*). Implementing a herbicide treatment to reduce buffel cover increased species richness and encourage higher grazing pressure showing animals were foraging in plots where buffel was removed (Melzer et al. 2014). The study suggests the removing buffel increased grasses available for wallabies and may be a viable management strategy to increase food availability for the wallabies. Nevertheless, challenges for buffel grass management include prolific seeding hence reinvasion, reduced grazing pressure by native species compared to cattle, and fire is only viable when controlled on a small scale (Department of Environment and Science 2022).

This study aimed to reassess the nutritional composition of plant species available within the habitat of the NHW, including invasive buffel grass, and how the nutritional composition of buffel grass compares to other taxa. The study also aimed to assess the seasonal variation in nutritional composition of plant species available at EFNP and RUNR. Determining the nutritional composition of forage available throughout the habitat of the NHW during all seasons will determine which species are nutrient dense, as well as their general digestibility. Comparing the nutritional composition of buffel grass to other forage species at EFNP and RUNR will provide insight into the potential role of buffel grass within the diet of the NHW. In addition, the study aimed to determine whether the nutritional quality of dietary items determines their abundance within the diet. Such insights will help to ensure the forage available at these sites and, more broadly, is managed appropriately (e.g. spraying invasive weeds), ensuring the NHW has access to adequate nutrition year‐round.

## 2 Methods

### 2.1 Study Sites

EFNP is a 2750 ha area of eucalypt woodland within the Brigalow Belt, in central Queensland (22°21′ S, 146°41′ E), in the grassland vegetation climate zone at an elevation of 230 m (Department of Environment and Science 2022). EFNP consists of deep alluvial sand deposits along a creek with Clarkson's bloodwood (*Corymbia clarksoniana*), Moreton Bay ash (*Corymbia tessellaris*) and Reid River box (*Eucalyptus brownii*), the main tree species present. Dominant grass species include white spear grasses (*Aristida* spp.), bottle‐washer grasses (*Enneapogon* spp.) and golden beardgrass (*Chrysopogon fallax*), although more than 20 native grass species were documented in 1998 (Woolnough 1998). Rainfall at EFNP is extremely variable (Figure S1), and there is a high frequency of drought; thus, park vegetation is constantly changing (Woolnough 1998). RUNR is located at Yarran Downs near St George in southern Queensland (27°56′ S, 148°52′ E) and is approximately 130 ha of eucalypt woodland in the subtropical vegetation climate zone (Department of Environment and Science 2022). The dominant vegetation includes silver‐leaved ironbark (*Eucalyptus melanophloia*), poplar box (*Eucalyptus populnea*), white cypress pine (*Callitris columellaris*) and belah (*Casuarina cristata*). The ground cover consists of grasses, such as mulga mitchell grass (*Thyridolepis mitchelliana*) and feathertop wiregrass (*Aristida latifolia*) (Jorgensen 2017). While the two sites are categorised within different vegetation climate zones, they are within the same seasonal rainfall zone which is wet summers and low winter rainfall. The distance between EFNP and RUNR is approximately 600 km.

### 2.2 Plant and Faecal Sample Collection

Plant (leaf and stem) and scat samples (100–200 g) were collected from both EFNP and RUNR to assess nutrient composition across both NHW habitats. Plant and scat samples were collected during four seasons at EFNP: winter and spring 2020, summer 2020/21 and winter 2021; and during six seasons at RUNR: winter and spring 2020, summer 2020/21 and autumn, winter and spring 2021. Plant and faecal samples were collected from near burrow entrances. Plants were collected from this region as southern hairy‐nosed wombats graze in ‘halos’ near their warrens (Lehmann 1979), and it is possible that NHW have a similar feeding strategy.

Plants were selected if the taxon had been identified as a NHW dietary item in previous studies (Crossman 1988; Flosser 1986; Woolnough 1998) or if the taxon was frequently encountered by the sampler. Forage composition and frequency data was not collected; hence, the frequency of occurrence of each taxon was based on the sampler's interpretation. Scats were selected based on freshness, as indicated by colour and texture, as they have previously been used in other studies using scats (Banks et al. 2002; Old, Hermsen, and Young 2020). The location of each plant and scat sample was recorded using a GPS (Tables S1–S4).

All samples were stored in paper bags at −20°C until analysis. The genus and species of each plant sample was identified using online databases ‘JSTOR Global Plants’ (JSTOR 2023). Plant samples that could not be identified were sent to the Queensland Herbarium for identification. The genus of all samples was identified, and the species was identified where possible. All samples were weighed and dried in an oven at 60°C for over 24 h until they had reached a constant mass and then re‐weighed and ground.

### 2.3 Nutritional Analysis

The nutritional composition of scat and plant samples was assessed. For plant samples, values of nitrogen (N), gross energy (GE), acid detergent fibre (ADF), neutral detergent fibre (NDF) and ash were determined, as well as individual minerals. For scat samples, N and GE values were determined.

Nitrogen content was determined using an ELEMENTAR vario EL cube (Elementar Australia Pty Ltd., Sydney, Australia), and crude protein was calculated as N × 6.25. Gross energy (GE) was determined using an oxygen bomb calorimeter (Parr 6200, Parr Instrument Co, Moline, IL) with a benzoic acid standard. NDF and ADF were determined using the Van Soest method which uses chemical extraction followed by gravimetric determination of the fibre residue (Van Soest 2020a, 2020b). Mineral composition (Al, C, C, Ca, Co, Cr, Cu, Fe, K, Mg, Mn, Na, Ni, O, S and Zn) of each plant sample was determined by microwave digest and ICP‐AES analysis at the Department of Primary Industries, Wollongbar, NSW.

### 2.4 Statistical Analysis

Statistical analyses were performed using R version 4.0.4 (R Core Team 2022). Factorial analyses of variance (ANOVA) were performed to determine whether site, season or genus affected the GE, protein, ADF, NDF, ash or minerals in plant samples. Plant genus was used as an independent variable instead of species, as accurate identification of species was not possible for all samples. Site‐specific two‐way ANOVAs were performed to determine whether season and or genus affected the GE, protein, ADF, NDF, ash or minerals in plants within each site. These site‐specific ANOVAs were performed in addition to the factorial ANOVA to determine whether season or genus affected nutritional factors within each individual site.

The relationship between the N present in scats (Nf) and the N present in plant samples (Np) was assessed. Nf was calculated as the mean N value of each scat assessed within a sampling period (sampling event of a single season at a single site), and Np was calculated as the mean N value of each plant assessed within a sampling period. Nf values were paired with the Np value for the corresponding sampling period (Table S7) and a linear regression was performed. The relationship between the GE in scats (GEf) and the GE in plant samples (GEp) was assessed using the same approach (Table S8).

### 2.5 Buffel Grass

Buffel grass was of particular interest within the study; thus, additional comparisons were made between buffel grass and other taxa. The GE, protein, ADF, NDF and ash of buffel grass were compared to the mean values of the remaining plants sampled during the corresponding sampling period. Buffel grass was sampled twice during winter 2020 at EFNP, and therefore, the mean nutritional values of the two samples were used for comparison.

### 2.6 Nutritional Composition Versus Dietary Abundance

To determine whether nutritional quality determined a food item's abundance within the diet, the nutritional values of forage items were matched to the abundance of those items in the diet. The occurrence and proportion of each plant in the scats were taken from Casey, Old, and Stannard (2023) during the corresponding sampling period. The proportions measure used was relative read abundance (RAA) from DNA analysis of wombat scats (Table S9).

$$\%{\mathrm{RRA}}_{i}=\frac{1}{N}\sum_{k=1}^{N}\frac{n_{i,k}}{\sum_{i=1}^{T}n_{i,k}}\times 100\%$$

where 𝑁 is the total number of faecal samples, *T* is the total number of species categories, *n*_*i,k*_ is the number of sequences of prey species *I* in sample *k*. The genus of all plant samples within this study was identified, but not the species; thus, the abundance of each genus within the diet was matched to each plant genus. Using R version 4.1.3 (R Core Team 2022), individual linear regressions were performed with dietary abundance as a function of each nutritional factor: plant GE, protein, ADF, NDF and ash values. Multiple regression was not appropriate due to multicollinearity between nutritional factors. Where a genus had been sampled more than once within a single sampling period, the values of each sample were averaged, and the mean value represented the genus.

## 3 Results

A total of 109 plant samples and 89 scat samples were collected: 39 plants (winter 2020 – 13, Spring 2020 – 7, summer 2020/21 – 10 and winter 2021 – 9) and 41 scats from EFNP and 70 plants (winter 2020 – 17, spring 2020 – 12, summer 2020/21 – 8, autumn 2021 – 12 and winter 2021 – 10, spring 2021 – 11) and 48 scats from RUNR (Tables S5 and S6). Sample collection did not occur at EFNP during autumn 2021 or spring 2021 due to COVID‐19 restrictions.

### 3.1 Site Differences

Factorial ANOVAs revealed that each of the five nutritional factors tested for in the plant samples (GE, protein, ADF, NDF and ash) differed significantly (*p* < 0.05) between the two sites. The mean GE, ADF and NDF content in samples was greater at EFNP than RUNR, but the mean ash in plant samples was greater at RUNR. There was greater range in seasonal mean plant protein values at EFNP (seasonal means: 3.31%–8.26%) compared to at RUNR (seasonal means: 4.71%–6.97%).

### 3.2 Gross Energy

**FIGURE 1:**
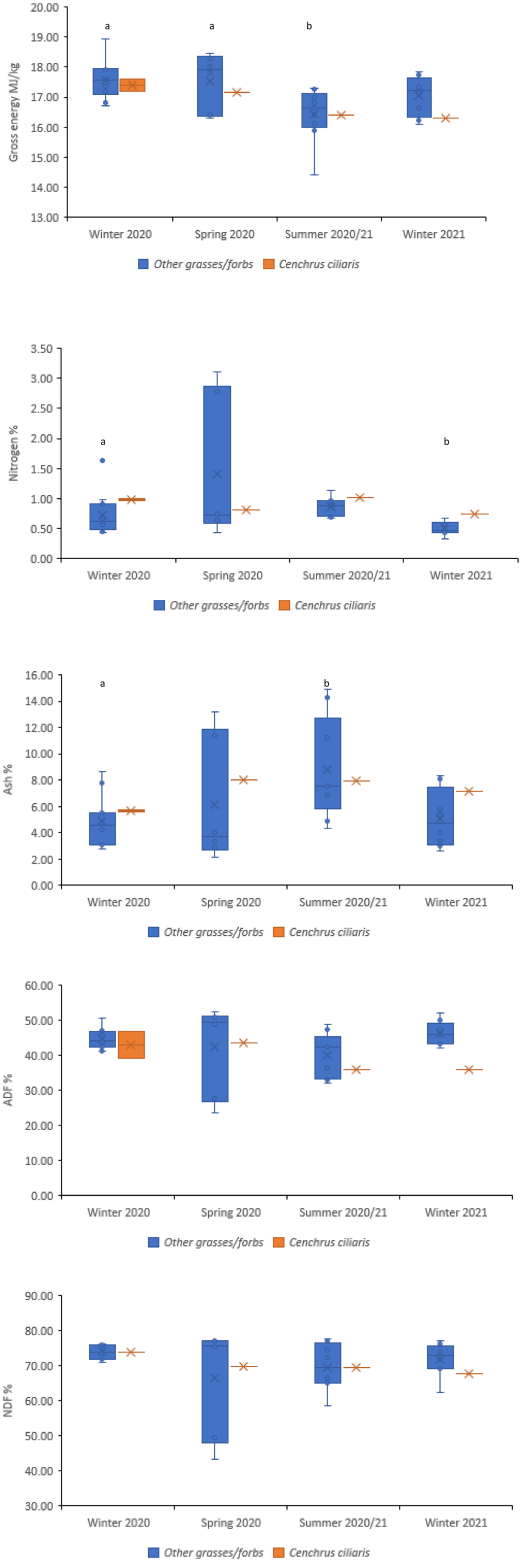
Gross energy (MJ/kg), nitrogen (%), ash (%), ADF (%) and NDF (%) values of *Cenchrus ciliaris* (buffel grass) during each sampling period at EFNP, compared to the mean protein, mean ash, mean GE, mean ADF and mean NDF values of the remaining samples within the corresponding sampling period (the seasonal mean excludes *Cenchrus ciliaris*). (a, b) significantly different from each other (*p* < 0.05).

Individually, season (*p* < 0.05) and genus (*p* < 0.05) significantly affected GE at EFNP, but there was no significant interaction between season and genus for GE (*p* > 0.05). Winter 2020 returned the highest mean GE (17.55 MJ/kg) and summer 2020/21 returned the lowest (16.43 MJ/kg; Figure 1). Some taxa, such as *Fimbristylis dichotoma*, returned comparably low GE values regardless of season, while other taxa such as *Enneapogon* spp. returned comparably high GE values across multiple seasons (Table S1).

At RUNR, there was a significant interaction between season and genus (*p* < 0.05) for GE; for example, *Panicum effusum*, *Enteropogon* spp. and *Themeda* spp. each returned GE values that were comparably high during one season, yet comparably low during another season (Table S2).

### 3.3 Protein

At EFNP, there was a significant interaction between season and genus (*p* < 0.05) for protein; for example, despite mean protein being greater in spring 2020 (8.26%) than winter 2020 (4.79%), the percentage of protein in buffel grass was greater during winter 2020 (6.25%; Figure 1) than in spring 2020 (5.07%; Figure 1; Table S1).

**FIGURE 2:**
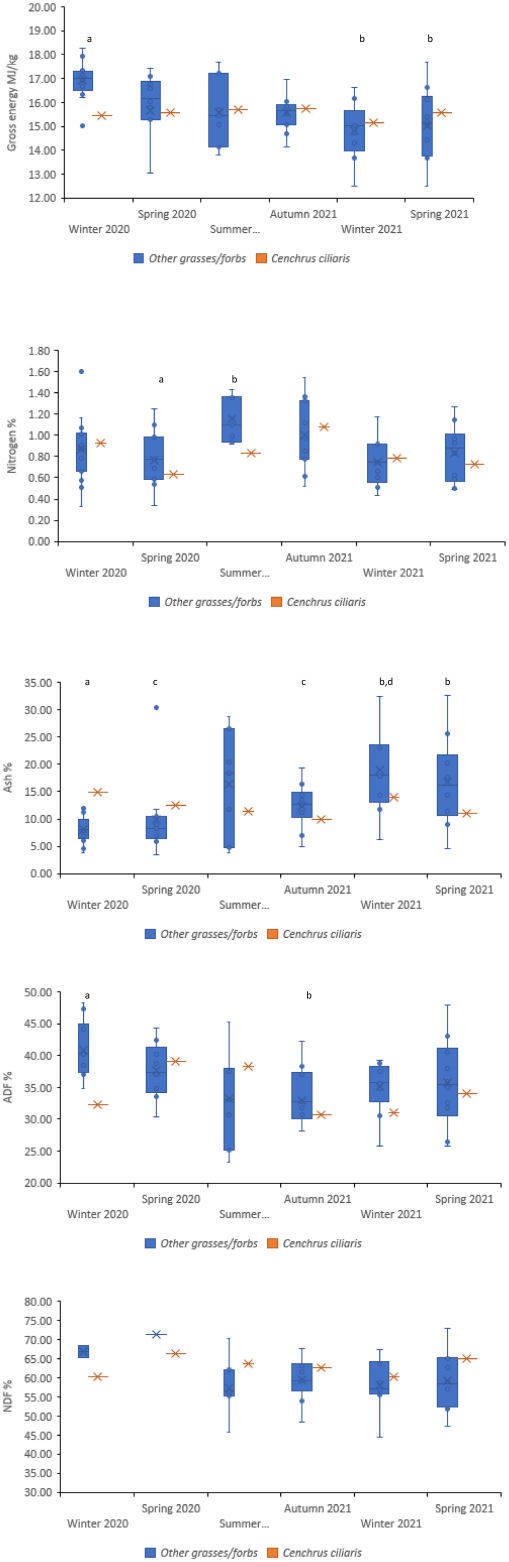
Gross energy (MJ/kg), nitrogen (%), ash (%), ADF (%) and NDF (%) values of *Cenchrus ciliaris* (buffel grass) during each sampling period at RUNR, compared to the mean protein, mean GE, mean ash, mean ADF and mean NDF values of the remaining samples within the corresponding sampling period (the seasonal mean excludes *Cenchrus ciliaris*). (a–d) significantly different from each other (*p* < 0.05).

There was no significant interaction between season and genus for protein at RUNR (*p* > 0.05); however, individually, season (*p* < 0.05) and genus (*p* < 0.05) significantly affected protein. Mean protein was highest during summer 2020/21 (6.97%) at RUNR and lowest during winter 2021 (4.71%; Figure 2). Some taxa returned comparably high or low protein values regardless of season, demonstrating the significant effect of genus on protein; for example, during spring 2020, autumn 2021 and winter 2021, *Paspalidium* spp. returned the highest protein value while *Themeda* spp. returned the lowest (Table S2).

### 3.4 Fibre

At RUNR, for ADF, there was a significant interaction of season and genus (*p* < 0.05); for example, *Enneapogon* spp., *Enteropogon* spp., *Themeda* spp. and buffel grass each returned a comparably low ADF value during at least one season and a comparably high ADF value during another season (Table S2). Mean ADF was highest during winter 2020 (40.12%) and lowest during autumn 2021 (32.68%; Figure 2). There were no significant effects of season (*p* > 0.05) or genus (*p* > 0.05) for ADF at EFNP. There were no significant effects of season (*p* > 0.05) or genus (*p* > 0.05) for NDF at either EFNP or RUNR.

### 3.5 Minerals

Ash was significantly affected by season at EFNP (*p* < 0.05). Mean ash was lowest during winter 2020 (4.99%) and winter 2021 (5.31%) and highest during summer 2020/21 (8.69%; Figure 1). Ash was significantly affected by an interaction of season and genus at RUNR (*p* < 0.05); for example, *Enteropogon* spp., *Eragrostis* spp. and *Themeda* spp. each returned a comparably low ash value during at least one season and a comparably high ash value during at least one other season (Table S2). Winter 2020 returned the lowest seasonal ash mean (8.54%), while winter 2021 returned the highest ash mean (18.45%), with ~10% difference (Figure 2).

Mineral content was significantly different between the two sites (*p* < 0.05) for Ca, Fe, K, Na. Ca, Fe and K in plant samples and was greater at RUNR compared to EFNP. Seasonal mean Ca values ranged from 0.17% to 0.31% at EFNP and from 0.22% to 0.38% at RUNR. Seasonal mean Fe values ranged from 112 mg/kg to 262 mg/kg at EFNP and from 287 mg/kg to 1946 mg/kg at RUNR. Seasonal mean K values ranged from 0.45% to 1.25% at EFNP to 0.49% to 1.35% at RUNR (Table 1). The range of seasonal mean Na values at RUNR (0.0037%–0.0122%) was greater than at EFNP (0.0063%–0.0096%; Table 1).

**TABLE 1:** Seasonal mean (min–max) mineral values of plants collected from Epping Forest National Park and Richard Underwood Nature Refuge.

Site	Season Units	*n*	Ca, %	Fe, mg/kg	K, %	Na, %	*p*, %
EFNP	Winter 2020	13	0.17 (0.09–0.41)	112 (62–280)	0.86 (0.37–2.3)	0.0068 (0.0024–0.023)	0.083 (0.045–0.16)
EFNP	Spring 2020	7	0.31 (0.07–0.79)	163 (89–330)	1.24 (0.12–4.1)	0.0063 (0.0024–0.014)	0.148 (0.058–0.33)
EFNP	Summer 20/21	10	0.23 (0.12–0.38)	262 (110–670)	1.25 (0.76–2.5)	0.0070 (0.0035–0.014)	0.115 (0.084–0.16)
EFNP	Winter 2021	9	0.22 (0.14–0.46)	160 (78–270)	0.45 (0.17–1.1)	0.0096 (0.0037–0.017)	0.073 (0.023–0.18)
RUNR	Winter 2020	6	0.22 (0.13–0.4)	332 (120–730)	0.85 (0.4–1.6)	0.0039 (0.0029–0.0056)	0.077 (0.033–0.2)
RUNR	Spring 2020	9	0.22 (0.15–0.35)	287 (42–650)	0.53 (0.18–1.2)	0.0047 (0.0005–0.0081)	0.061 (0.031–0.086)
RUNR	Summer 20/21	5	0.27 (0.13–0.43)	744 (160–2300)	0.69 (0.49–0.94)	0.0037 (0.0022–0.0061)	0.071 (0.043–0.092)
RUNR	Autumn 2021	5	0.23 (0.16–0.36)	1946 (370–6100)	1.35 (0.63–2.6)	0.0122 (0.0031–0.042)	0.106 (0.061–0.2)
RUNR	Winter 2021	6	0.38 (0.15–1.1)	1360 (390–2100)	0.69 (0.25–1.6)	0.0044 (0.002–0.013)	0.073 (0.047–0.12)
RUNR	Spring 2021	8	0.31 (0.12–0.98)	1176 (290–2000)	0.49 (0.18–1.1)	0.0050 (0.0033–0.012)	0.060 (0.036–0.095)

At EFNP, there was a significant interaction (*p* < 0.05) between season and genus for K and Na. Ca and P were significantly affected by season at EFNP (*p* < 0.05); Ca (0.31%) and P (0.148%) were highest during spring 2020, while Ca was lowest during winter 2020 (0.17%) and P was lowest during winter 2021 (0.073%; Table 1; Table S3). At RUNR, there was a significant interaction (*p* < 0.05) of season and genus for minerals: Ca, P and Na. For example, despite Na being lowest during summer 2020/21 (0.0037%) and highest during autumn 2021 (0.0122%; Table 1; Table S4). Fe and K were significantly affected by season (*p* < 0.05) at RUNR. Mean Fe was lowest during spring 2020 (287 mg/kg) and highest during autumn 2021 (1946 mg/kg), while mean K was highest during autumn 2021 (1.35%) and lowest during spring 2021 (0.49%; Table 1; Table S4).

### 3.6 Differences Between Individual Taxa at EFNP

At EFNP, buffel grass was sampled twice during winter 2020; one returned the lowest ADF value, while the other returned the third highest. During winter 2021, buffel grass returned the highest protein, lowest ADF and second lowest NDF value, but the third lowest GE (Table S1). Buffel grass returned the highest K and either the highest or second highest Mg values during winter 2020, summer 2020/21 and winter 2021 (Table S3). *Fimbristylis dichotoma* returned the highest protein and lowest ADF values during two of the four seasons sampled, as well as the lowest NDF and lowest GE values during three seasons (Table S1). *Fimbristylis dichotoma* returned the highest Ca value during each season at EFNP, and during spring 2020, returned the highest value of B, K, Mg and S, as well as the highest value of Co, Cu, Mn and Na of any sample collected from EFNP across all seasons (Table S3). During spring 2020, *Chrysopogon fallax* returned the second highest protein value and second lowest ADF, NDF and GE values (Table S1). *Enteropogon* spp. returned the highest GE and highest NDF values in both spring 2020 and summer 2020/21. During winter 2020, spring 2020 and summer 2020/21, *Enneapogon* spp. returned the second highest GE value as well as the second or third highest NDF value. During spring 2020, winter 2020 and winter 2021 at EFNP, *Aristida* spp. returned the lowest protein and highest ADF values as well as one of the three highest NDF values (Table S1). During these three seasons, *Aristida* spp. also returned the lowest Ca and Mg values as well as either the lowest or second lowest K value (Table S3).

### 3.7 Differences Between Individual Taxa at RUNR

At RUNR, buffel grass returned the lowest ADF and NDF values during winter 2020, the lowest NDF during spring 2020 and third lowest ADF during winter 2021 (Table S2). During summer 2020/21 at RUNR, buffel grass returned the lowest protein value and second highest ADF and NDF values. Buffel grass returned the highest or second highest Ca value during every season at RUNR except autumn, as well as the highest phosphorus (P) value during three seasons (Table S4). *Sporobolus caroli* was only sampled during winter 2021 and spring 2021, but during both seasons returned the lowest ADF values and the lowest and third lowest NDF values. Within the four seasons *Eragrostis* spp. were sampled, the taxon returned the second highest protein value during two seasons and one of the lowest two ADF values during three seasons. *Lomandra filiformis* returned either the highest or one of the highest protein values during five of the six sample periods at RUNR, as well as the highest GE value during four seasons, and the second and third highest during the remaining two seasons (Table S2). *Thyridolepis mitchelliana* returned one of the highest three protein values during four of the five seasons it was sampled (Table S2), as well as one of the lowest two Ca values during four seasons and the highest two Cr values during three seasons (Table S4). During spring 2020, autumn 2021 and winter 2021, *Paspalidium constrictum* returned the highest protein value, as well as the second lowest ADF value during spring 2020, but returned both the highest ADF and NDF value during summer 2020/21 and the highest NDF and third highest ADF during spring 2021. *Paspalidium constrictum* returned the lowest P value during three seasons (Table S4). *Themeda* spp. returned the lowest protein value, and one of the second or third highest ADF values during three of the four seasons it was sampled. *Aristida* spp. and *Panicum effusum* both frequently returned one of the lowest three protein values and one of the highest three ADF or NDF values per sampling period at RUNR (Table S2). *Aristida* spp. returned one of the lowest two Ca values during three seasons, while *Panicum effusum* returned the highest Na and Zn in autumn, winter and spring 2021, and either the highest or second highest P during three seasons (Table S4).

### 3.8 Buffel Grass Versus Other Taxa

At EFNP, buffel grass contained more protein than the corresponding seasonal mean (all other plants collected) during winter 2020, summer 2020/21 and winter 2021, as well as lower ADF and NDF during these three seasons (Figure 1). The GE in buffel grass was lower than the seasonal mean in all four seasons; however, the values were very similar. During winter 2020 and winter 2021, the ash content of buffel grass was lower than the seasonal mean but higher in summer 2020/21 and spring 2020 (Figure 1).

At RUNR, buffel grass contained more protein than the seasonal mean during winter 2020, autumn 2021 and winter 2021 but less protein than the seasonal mean during spring 2020, summer 2020/21 and spring 2021 (Figure 2). Buffel contained less ADF than the seasonal mean during four of six seasons while the seasonal mean for NDF was less than that of buffel grass during four seasons. GE was higher in buffel grass during four of six seasons at RUNR, but the differences were small (Figure 2). Ash was lower in buffel grass during four of six seasons, with considerable differences between buffel grass and seasonal means (Figure 2).

### 3.9 Faecal Nitrogen and Gross Energy

**FIGURE 3:**
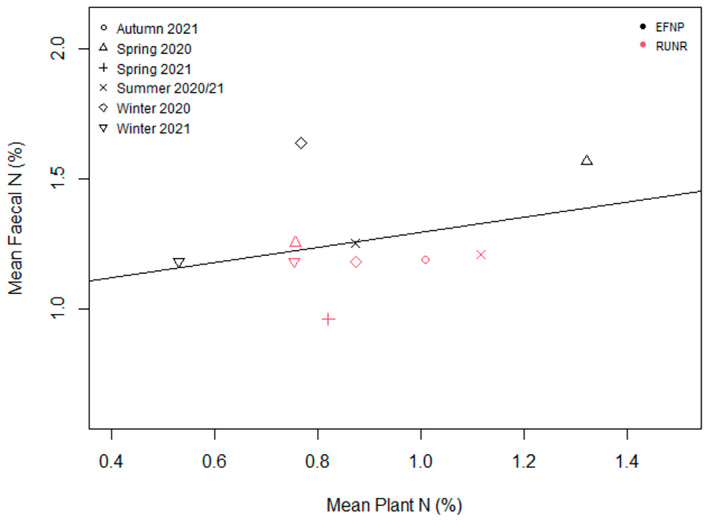
The relationship between the mean N (%) available within plants and the mean N (%) in northern hairy‐nosed wombat faeces during each sampling period. There was no significant relationship between Nf and Np (*p* > 0.05).

**FIGURE 4:**
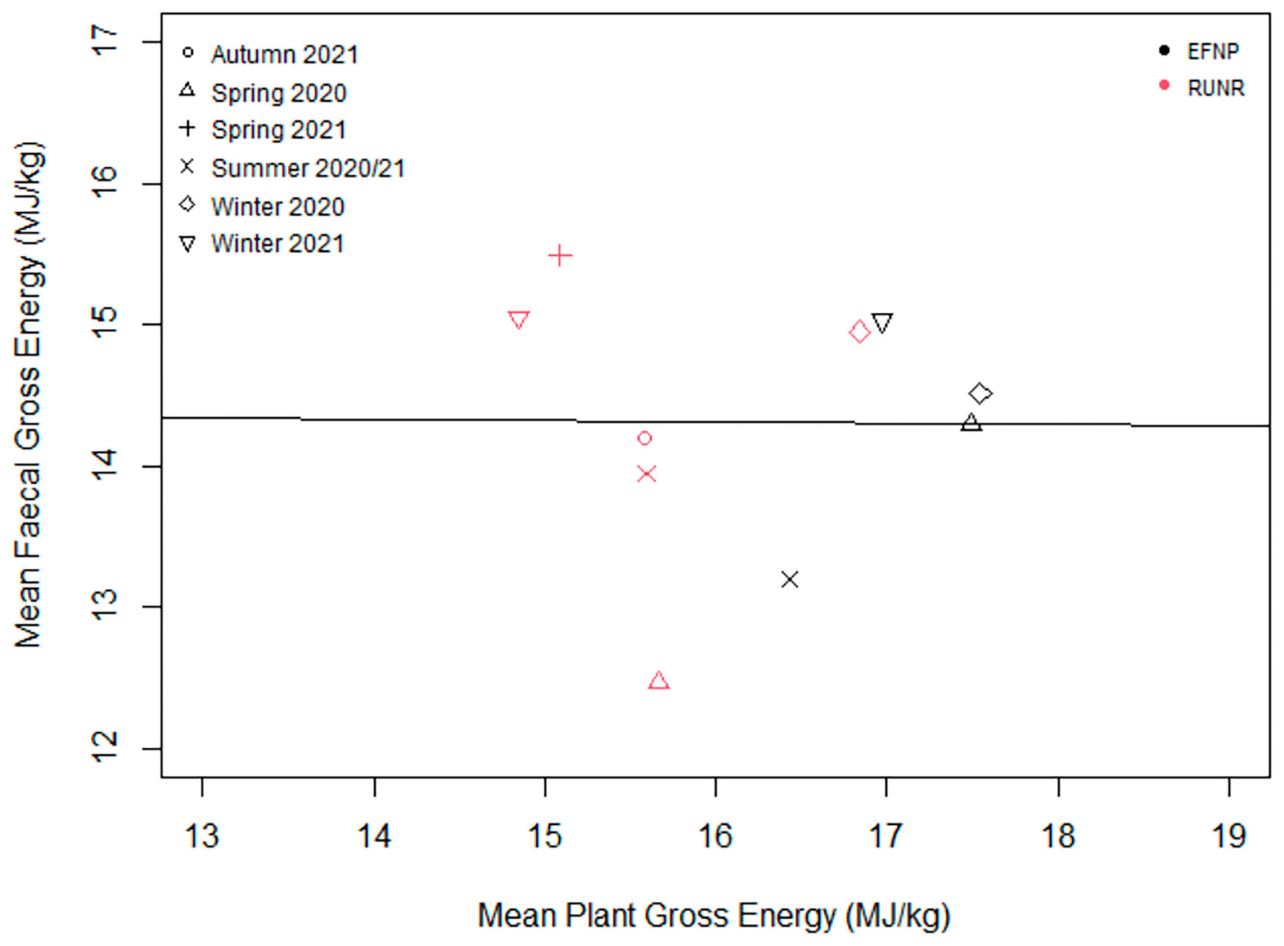
The relationship between the mean gross energy (MJ/kg) available within plants and the mean gross energy (MJ/kg) in northern hairy‐nosed wombat faeces during each sampling period. There was no significant relationship between GEf and GEp (*p* > 0.05).

There was no significant relationship between Nf and Np (*p* > 0.05; Figure 3), nor was there a significant relationship between GEf and GEp (*p* > 0.05; Figure 4).

### 3.10 Nutritional Composition Versus Dietary Abundance

Using the RAA from Casey, Old, and Stannard (2023) as a measure for abundance of each plant species identified in the wombat scats, linear regressions revealed that there was no significant relationship between the amount of GE, protein, ADF, NDF or ash in plants and the abundance of those plants in the diet.

## 4 Discussion

Despite numbers of NHWs steadily increasing at the EFNP site since the 1980s, and a second translocation site established, they remain critically endangered (Taggart, Martin, and Horsup 2016). There is very limited knowledge of the population dynamics due to their cryptic and skittish nature; however, no easily discernible differences have been observed in the NHWs at the two sites (Department of Environment and Science 2022). Management of the species is largely ‘hands‐off’ and methods to support the populations are largely non‐invasive. For this reason, this study aimed to investigate the nutrition requirements of the NHW using non‐invasive techniques, by analysing NHW scat and plant species available as forage in their habitat.

The nutritional content of forage available to the NHW at EFNP and RUNR was determined and found to vary significantly depending on site, season and plant genus. The nutrient content available to the NHW at each site was significantly different for GE, protein, ADF, NDF and ash; thus, nutrient levels available to each wombat population differ. GE, protein and ash at both sites, and ADF at RUNR, were significantly affected by season. The significant interactions between season and genus for several nutrients at both EFNP and RUNR suggest that each taxon's role in the diet of the NHW varies seasonally.

Overall, the forage available to the NHW at RUNR contained less fibre (ADF and NDF) and more protein than the forage available at EFNP, suggesting that the forage available to the NHW at RUNR would be more digestible due to the lower levels of fibre, given the fibrous content in forage largely determines its digestibility (Barboza 1993; Camp et al. 2015). Similar to our results for fibre, grasses within southern hairy‐nosed wombat habitat showed seasonal variation in ADF levels with concentrations of 22.7%–58.1% (Wells 1973). Previous studies in bare‐nosed and southern hairy‐nosed wombat has shown digestible energy to be 39% for a diet with NDF of 66.3% (Barboza, Hume, and Nolan 1993). If digestible energy for NHW is similar to that described for other wombats in Barboza, Hume, and Nolan (1993), the approximate digestibly energy of buffel grass would be 6.3 kJ/g (Tables S1 and S2). Maintenance energy requirements are 140 kJ/kg^0.75^; thus, a 30 kg wombat would need 1795 kJ per day, which would equate to a daily intake of 285 g of dry buffel grass. The buffel grass should therefore exceed 0.99%N to meet the maintenance nitrogen requirements for a wombat (201 mg N kg^−0.75^ d^−1^ or 2.58 g N d^−1^ for a 30 kg wombat). In most seasons, at both sites, buffel grass is below the nitrogen requirement of a 30 kg wombat. Hence, a wombat would be required to consume more buffel grass to meet their needs and/or consume other species of grasses. This is consistent with the finding of Casey, Old, and Stannard (2023) that while NHW are consuming mostly buffel grass at both sites they are also consuming other grasses, which would contribute to their nitrogen intake.

As well as fibre (ADF and NDF), nitrogen is used to estimate forage quality (Loeb, Schwab, and Demment 1991; Owen‐Smith 1982). Based on the mean seasonal values of nitrogen, ADF and NDF, forage quality was highest in spring at EFNP. At RUNR, winter and spring had the poorest quality and highest quality during summer 2020/21 and autumn 2021. Nitrogen was variable across seasons and sites, as well as taxa, with some species of grass having high levels of protein, such as *Fimbristylis dichotoma* (3.11%) which is consumed by NHW (Casey, Old, and Stannard 2023). To meet maintenance nitrogen requirements, a 30 kg wombat would only require a daily dry matter intake of 83 g of *Fimbristylis dichotoma*, but it would not meet dry matter or energy requirements. Similarly, forage within southern hairy‐nosed wombat habitats have seasonal variation in nitrogen levels with concentrations of 0.85%–2.61% (Wells 1973).

While specific mineral requirements are not known for NHW, foraging habits are likely influenced by season, reproductive status and sex. Diet choice is influenced by seasonal changes (Casey, Old, and Stannard 2023) and the availability of food as well as its nutrient composition. Furthermore, while there were differences in mineral composition across both sites in this study, there were also significant seasonal differences in minerals at each site. At EFNP, Ca and P were significantly higher in spring, and at RUNR, Fe and K were significantly higher in autumn. Ca, P and K are important for bone formation and nerve and muscle function; Fe is important for building oxygen stores in the body (Barboza, Parker, and Hume 2009). Cu toxicity has previously been reported in one southern hairy‐nosed wombat (Barboza and Vanselow 1990); hence, they presumably require less Cu than eutherian species. No toxicity has been reported or observed in NHWs.

A major determinant of pasture growth in EFNP is summer rainfall (Johnson 1991). Rainfall during our study period was relatively low in the months of sampling and during the preceding 30 days prior to each sampling (Figure S1). Total rainfall was ≤ 65 mm per month during the sampling months at EFNP and ≤ 50 mm per month during the sampling months at RUNR. Hence, pasture growth would be expected to be low to moderate during our sampling periods, and protein levels would therefore also remain relatively low. Further studies should collect plants following high rainfall during growth periods to assess nutrients available as these higher rainfalls, particularly during summer, coincide with increased breeding in NHW (Crossman, Johnson, and Horsup 1994).

Previous studies of the nutritional intake of herbivorous mammals have found a significant relationship between faecal N and forage or dietary N (Gil‐Jimenez et al. 2015; Leslie, Bowyer, and Jenks 2008; Wrench, Meissner, and Grant 1997), as well as between faecal GE and forage or dietary GE (Carpio et al. 2015; Gil‐Jimenez et al. 2015; Ueno et al. 2007). There are multiple factors that may have contributed to the lack of significant correlation between plant and faecal values in this study, including inadequate sample size or that the plants contributing to the Np and GEp values were not contributing to the diet. Future research should consider the inclusion of a more thorough investigation of these relationships, as the determination of predictive equations for forage content from scats provides opportunity for low‐cost monitoring of forage quality. A minimum threshold of crude protein (5%) is required for grazers (Robbins 1983). A lower crude protein intake causes nutritional stress (Robbins 1983). Mean faecal protein values in the wombats were above 5% in all seasons across both sites, suggesting that they were consuming nutritionally adequate diets.

There was no significant linear relationship between nutritional values of taxa and the abundance of those taxa in the diet, which supports the findings of Woolnough (1998) and Flosser (1986) that the NHW is a generalist feeder with a diet that reflects the availability of forage. Woolnough (1998) found that the NHW consumed plant species at a similar frequency to which they occurred within the habitat, and that changes in the diet of the NHW did not correlate with changes in forage nutritive quality. Flosser (1986) concluded that the NHW was likely a generalist feeder, despite preferring to consume particular plant species; however, we could not confirm this conclusion as forage availability was not quantified. An assessment of forage availability (composition, species frequency and biomass) within NHW habitat would facilitate investigating these previous suggestions that the diet of the NHW is determined by forage availability. Furthermore, while NHWs have not been recorded to consume other non‐dietary components for nutrient uptake, such as through geophagy, this may be possible and requires further investigation.

This study successfully provided an updated assessment of the nutritional quality of forage available to the NHW, which will assist in management and conservation of the species; however, the reliability of results could have been improved with more balanced sampling. The same number of samples were not collected at each site or during each season, and there was a lack of consistency in which plant species or genera were collected in each sampling period. In addition, the stage of plant life cycle was not controlled for in sampling, despite the nutritional quality of forage items changing with age (Modi 2007). The reliability of the comparisons made in this study of nutritional availability between sites, seasons and plant taxa may therefore be limited. However, certain factors likely limited the opportunity to achieve balanced sampling, for example, sampling was partially driven by taxa frequency which may have changed temporally, but more reliable results could be achieved in future with improved sampling.

The significant differences in the nutritional content of forage between the two sites confirmed that the nutritional availability of the two NHW populations is different and thus require individual site‐specific management. Within each site, the nutritional quality of individual taxa as determined in this study should inform decisions pertaining to the management of the vegetation community. The nutritional quality of buffel grass in comparison to other potential dietary items should inform the plant species' management. As an invasive species, buffel grass poses a threat to the vegetation community at both EFNP and RUNR; however, it is has become a major dietary item of the NHW, particularly during winter when forage is poorest and buffel grass offers relatively improved nutritional quality. No digestibility studies have been undertaken for NHW, and it would be ideal to conduct digestibility experiments to further understand the nutrient availability within the food consumed by wombats. In the longer term, information obtained from this study will also assist in informing the selection of potential new translocation sites based on nutritional composition of food items within potential habitats.

## Author Contributions

**Fiona F. Casey:** data curation (equal), formal analysis (equal), methodology (equal), writing – original draft (equal), writing – review and editing (equal). **Julie M. Old:** conceptualization (equal), funding acquisition (equal), methodology (equal), project administration (equal), supervision (equal), writing – review and editing (equal). **Hayley J. Stannard:** conceptualization (equal), funding acquisition (equal), methodology (equal), project administration (equal), supervision (equal), writing – review and editing (equal).

## Conflicts of Interest

The authors declare no conflicts of interest.

## Supporting information


Appendix S1.

## Data Availability

The datasets generated during and/or analysed during the current study are available in Appendix S1.
