# Supplementary material for: Nutritional Composition of Forage Available to the Northern Hairy‐Nosed Wombat
Source: Ecol Evol. 2024 Nov 7;14(11):e70514. doi: 10.1002/ece3.70514 (PMC11542933; doi:10.1002/ece3.70514)
Supplement: Supplementary file 1 — Appendix S1. [file ECE3-14-e70514-s001.docx]

Appendix

Table S1. Epping Forest National Park (EFNP) plant sample nutrition values and nitrogen and energy requirements.

| **Site** | **Season** | **Genus** | **Species** | **Collection date** | **Latitude** | **Longitude** | **GE (kJ/g)** | **N (%)** | **Protein (%)** | **ADF (%)** | **NDF (%)** | **Ash (%)** | **Organic matter (%)** | **Digestible energy (kJ/g)^a^** | **DMI required to meet MNR (g/d)^b^** | **DMI required to meet MER (g/d)^c^** |  |
| --- | --- | --- | --- | --- | --- | --- | --- | --- | --- | --- | --- | --- | --- | --- | --- | --- | --- |
|  |  |  |  |  |  |  |  |  |  |  |  |  |  |  |  |  |  |
| EFNP | Winter 2020 | *Aristida* | NA | 4/06/2020 | -22.3824 | 146.7038 | 17.56 | 0.68 | 4.25 | 47.11 | - | 3.11 | 96.89 | 6.85 | 379.4 | 262.0 |  |
| EFNP | Winter 2020 | *Aristida* | NA | 4/06/2020 | -22.3727 | 146.6916 | 17.91 | 0.44 | 2.75 | 50.5 | 75.85 | 3.38 | 96.62 | 6.98 | 586.4 | 257.2 |  |
| EFNP | Winter 2020 | *Cenchrus* | *ciliaris* | 4/06/2020 | -22.3824 | 146.7039 | 17.21 | 1 | 6.25 | 39.19 | - | 5.63 | 94.37 | 6.71 | 258.0 | 267.5 |  |
| EFNP | Winter 2020 | *Cenchrus* | *ciliaris* | 4/06/2020 | -22.3828 | 146.7044 | 17.61 | 0.97 | 6.07 | 46.8 | 73.96 | 5.74 | 94.26 | 6.87 | 266.0 | 261.3 |  |
| EFNP | Winter 2020 | *Chrysopogon* | *fallax* | 4/06/2020 | -22.3851 | 146.7045 | 17.56 | 0.92 | 5.76 | 44.29 | 73.99 | 4.72 | 95.28 | 6.85 | 280.4 | 262.0 |  |
| EFNP | Winter 2020 | *Digitaria* | NA | 4/06/2020 | -22.3724 | 146.696 | 16.82 | 0.49 | 3.09 | 41.17 | - | 7.81 | 92.19 | 6.56 | 526.5 | 273.6 |  |
| EFNP | Winter 2020 | *Enneapogon* | NA | 4/06/2020 | -22.3843 | 146.7052 | 18 | 0.68 | 4.28 | 46.58 | 75.8 | 3.12 | 96.88 | 7.02 | 379.4 | 255.7 |  |
| EFNP | Winter 2020 | *Enteropogon* | *ramosus* | 4/06/2020 | -22.385 | 146.705 | 17.56 | 0.63 | 3.95 | 46.77 | 73.49 | 4.54 | 95.46 | 6.85 | 409.5 | 262.0 |  |
| EFNP | Winter 2020 | *Eragrostis* | NA | 4/06/2020 | -22.3725 | 146.696 | 17.67 | 0.45 | 2.84 | 45.7 | - | 4.29 | 95.71 | 6.89 | 573.3 | 260.5 |  |
| EFNP | Winter 2020 | *Fimbristylis* | *dichotoma* | 4/06/2020 | -22.3844 | 146.7052 | 16.71 | 1.63 | 10.19 | 44.14 | - | 8.68 | 91.32 | 6.52 | 158.3 | 275.3 |  |
| EFNP | Winter 2020 | *Heteropogon* | *contortus* | 4/06/2020 | -22.3681 | 146.691 | 17.18 | 0.58 | 3.62 | 41.27 | 71.02 | 5.55 | 94.45 | 6.7 | 444.8 | 267.9 |  |
| EFNP | Winter 2020 | *Themeda* | *triandra* | 4/06/2020 | -22.3681 | 146.691 | 17.38 | 0.49 | 3.04 | 43.93 | 71.79 | 5.5 | 94.5 | 6.78 | 526.5 | 264.7 |  |
| EFNP | Winter 2020 | *Triodia* | *mitchellii* | 4/06/2020 | -22.3727 | 146.6908 | 18.94 | 0.98 | 6.14 | 42.48 | 76.32 | 2.79 | 97.21 | 7.39 | 263.3 | 242.9 |  |
| EFNP | Spring 2020 | *Aristida* | NA | 3/11/2020 | -22.3854 | 146.7055 | 18.01 | 0.44 | 2.72 | 52.31 | 77.02 | 2.19 | 97.81 | 7.02 | 586.4 | 255.7 |  |
| EFNP | Spring 2020 | *Cenchrus* | *ciliaris* | 3/11/2020 | -22.3829 | 146.7038 | 17.15 | 0.81 | 5.07 | 43.49 | 69.73 | 8 | 92 | 6.69 | 318.5 | 268.3 |  |
| EFNP | Spring 2020 | *Chrysopogon* | *fallax* | 3/11/2020 | -22.3854 | 146.7054 | 16.4 | 2.78 | 17.4 | 27.86 | 49.49 | 11.39 | 88.61 | 6.4 | 92.8 | 280.5 |  |
| EFNP | Spring 2020 | *Enneapogon* | NA | 3/11/2020 | -22.3846 | 146.7054 | 18.3 | 0.65 | 4.06 | 50.93 | 76.05 | 3.36 | 96.64 | 7.14 | 396.9 | 251.4 |  |
| EFNP | Spring 2020 | *Enneapogon* | NA | 3/11/2020 | -22.3841 | 146.7044 | 17.8 | 0.71 | 4.46 | 49.01 | 75.4 | 2.87 | 97.13 | 6.94 | 363.4 | 258.6 |  |
| EFNP | Spring 2020 | *Enteropogon* | *ramosus* | 3/11/2020 | -22.3838 | 146.7045 | 18.47 | 0.75 | 4.67 | 50.12 | 77.16 | 4.02 | 95.98 | 7.2 | 344.0 | 249.3 |  |
| EFNP | Spring 2020 | *Fimbristylis* | *dichotoma* | 3/11/2020 | -22.3849 | 146.7056 | 16.3 | 3.11 | 19.45 | 23.51 | 43.28 | 13.17 | 86.83 | 6.36 | 83.0 | 282.2 |  |
| EFNP | Summer 2020/21 | *Aristida* | NA | 26/02/2021 | -22.3839 | 146.7036 | 16.81 | 0.74 | 4.62 | 42.28 | 72.39 | 6.83 | 93.17 | 6.56 | 348.6 | 273.6 |  |
| EFNP | Summer 2020/21 | *Cenchrus* | *ciliaris* | 26/02/2021 | -22.3831 | 146.7039 | 16.42 | 1.01 | 6.34 | 36.05 | 69.56 | 7.97 | 92.03 | 6.4 | 255.4 | 280.5 |  |
| EFNP | Summer 2020/21 | *Chrysopogon* | *fallax* | 26/02/2021 | -22.385 | 146.705 | 16.14 | 1.14 | 7.12 | 33.9 | 66.42 | 11.22 | 88.78 | 6.29 | 226.3 | 285.4 |  |
| EFNP | Summer 2020/21 | *Chrysopogon* | *fallax* | 26/02/2021 | -22.385 | 146.7051 | 16.65 | 0.87 | 5.46 | 42.27 | - | 7.84 | 92.16 | 6.49 | 296.6 | 276.6 |  |
| EFNP | Summer 2020/21 | *Enneapogon* | *robustissimus* | 26/02/2021 | -22.384 | 146.7049 | 17.26 | 0.73 | 4.56 | 48.81 | 77.25 | 4.33 | 95.67 | 6.73 | 353.4 | 266.7 |  |
| EFNP | Summer 2020/21 | *Enneapogon* | *polyphyllus* | 26/02/2021 | -22.3849 | 146.7043 | 15.88 | 0.98 | 6.1 | 36.47 | 64.97 | 14.9 | 85.1 | 6.19 | 263.3 | 290.0 |  |
| EFNP | Summer 2020/21 | *Enteropogon* | NA | 26/02/2021 | -22.3846 | 146.7042 | 17.3 | 0.69 | 4.31 | 47.48 | 77.68 | 4.86 | 95.14 | 6.75 | 373.9 | 265.9 |  |
| EFNP | Summer 2020/21 | *Eragrostis* | NA | 26/02/2021 | -22.385 | 146.705 | 16.98 | 0.68 | 4.26 | 43.17 | 74.41 | 7.09 | 92.91 | 6.62 | 379.4 | 271.1 |  |
| EFNP | Summer 2020/21 | *Fimbristylis* | *dichotoma* | 26/02/2021 | -22.3831 | 146.704 | 16.41 | 0.96 | 6.01 | 32.9 | 58.66 | 7.58 | 92.42 | 6.4 | 268.8 | 280.5 |  |
| EFNP | Summer 2020/21 | *Perotis* | *rara* | 26/02/2021 | -22.3846 | 146.7055 | 14.41 | 0.91 | 5.71 | 32.16 | 65.18 | 14.27 | 85.73 | 5.62 | 283.5 | 319.4 |  |
| EFNP | Winter 2021 | *Aristida* | NA | 29/08/2021 | -22.3731 | 146.6933 | 17.35 | 0.46 | 2.88 | 52.09 | 77.24 | 2.63 | 97.37 | 6.77 | 560.9 | 265.1 |  |
| EFNP | Winter 2021 | *Aristida* | NA | 29/08/2021 | -22.3705 | 146.6896 | 17.15 | 0.33 | 2.04 | 49.93 | 76.31 | 3.4 | 96.6 | 6.69 | 781.8 | 268.3 |  |
| EFNP | Winter 2021 | *Cenchrus* | *ciliaris* | 29/08/2021 | -22.3794 | 146.6966 | 16.3 | 0.75 | 4.68 | 35.93 | 67.72 | 7.14 | 92.86 | 6.36 | 344.0 | 282.2 |  |
| EFNP | Winter 2021 | *Chrysopogon* | *fallax* | 29/08/2021 | -22.3784 | 146.698 | 16.25 | 0.67 | 4.17 | 43.23 | 69.49 | 8.35 | 91.65 | 6.34 | 385.1 | 283.1 |  |
| EFNP | Winter 2021 | *Enneapogon* | NA | 29/08/2021 | -22.3796 | 146.6982 | 17.33 | 0.5 | 3.11 | 47.27 | 74.02 | 3.02 | 96.98 | 6.76 | 516.0 | 265.5 |  |
| EFNP | Winter 2021 | *Eragrostis* | NA | 29/08/2021 | -22.3789 | 146.6986 | 17.75 | 0.45 | 2.8 | 46.25 | 73.9 | 5.37 | 94.63 | 6.92 | 573.3 | 259.4 |  |
| EFNP | Winter 2021 | *Fimbristylis* | *dichotoma* | 29/08/2021 | -22.3735 | 146.6929 | 16.09 | 0.62 | 3.87 | 45.81 | 62.31 | 8.07 | 91.93 | 6.28 | 416.1 | 285.8 |  |
| EFNP | Winter 2021 | *Heteropogon* | *contortus* | 29/08/2021 | -22.3784 | 146.698 | 16.66 | 0.43 | 2.69 | 43.91 | 69.11 | 5.77 | 94.23 | 6.5 | 600.0 | 276.2 |  |
| EFNP | Winter 2021 | *Triodia* | *mitchellii* | 29/08/2021 | -22.3705 | 146.6898 | 17.84 | 0.58 | 3.6 | 42.06 | 71.84 | 4.04 | 95.96 | 6.96 | 444.8 | 257.9 |  |

NA – Identified to Genus level only. - sample size too small to complete analysis. ^a^ Digestible energy calculated using 39% as approximation. ^b^ Daily dry matter intake (DMI) required to meet maintenance nitrogen requirement (MNR) for a 30kg wombat. ^c^ Daily dry matter intake (DMI) required to meet maintenance energy (MER) requirement for a 30kg wombat.

Table S2. Richard Underwood Nature Refuge (RUNR) plant sample nutrition values and nitrogen and energy requirements.

| **Site** | **Season** | ***Genus*** | **Species** | **Collection date** | **Latitude** | **Longitude** | **GE (kJ/g)** | **N (%)** | **Protein (%)** | **ADF (%)** | **NDF (%)** | **Ash (%)** | **Organic matter (%)** | **Digestible energy (kJ/g)^a^** | **DMI required to meet MNR (g/d)^b^** | **DMI required to meet MER (g/d)^c^** |
| --- | --- | --- | --- | --- | --- | --- | --- | --- | --- | --- | --- | --- | --- | --- | --- | --- |
| RUNR | Winter 2020 | *Aristida* | NA | 2/07/2020 | -27.664997 | 148.69798 | 16.43 | 0.81 | 5.08 | 38.5 | - | 11.94 | 88.06 | 6.41 | 318.5 | 280.0 |
| RUNR | Winter 2020 | *Aristida* | NA | 2/07/2020 | -27.662261 | 148.698305 | 16.8 | 0.8 | 4.97 | 37.42 | - | 7.97 | 92.03 | 6.55 | 322.5 | 274.0 |
| RUNR | Winter 2020 | *Bothriochloa* | *macra* | 2/07/2020 | -27.66248 | 148.696833 | 17.14 | 0.66 | 4.11 | 40.18 | - | 8.46 | 91.54 | 6.68 | 390.9 | 268.7 |
| RUNR | Winter 2020 | *Cenchrus* | *ciliaris* | 2/07/2020 | -27.664997 | 148.69798 | 15.45 | 0.93 | 5.79 | 32.27 | 60.36 | 14.97 | 85.03 | 6.03 | 277.4 | 297.7 |
| RUNR | Winter 2020 | *Chrysopogon* | *fallax* | 2/07/2020 | -27.66763 | 148.70219 | 17.43 | 1.17 | 7.28 | 40.51 | 68.55 | 6.73 | 93.27 | 6.8 | 220.5 | 264.0 |
| RUNR | Winter 2020 | *Enneapogon* | NA | 2/07/2020 | -27.66763 | 148.70219 | 17.08 | 0.91 | 5.68 | - | - | - | - | 6.66 | 283.5 | 269.5 |
| RUNR | Winter 2020 | *Enneapogon* | NA | 2/07/2020 | -27.66763 | 148.70219 | 16.7 | 0.92 | 5.77 | 48.26 | 65.31 | 8.59 | 91.41 | 6.51 | 280.4 | 275.7 |
| RUNR | Winter 2020 | *Enteropogon* | *unispiceus* | 2/07/2020 | -27.66763 | 148.70219 | 16.19 | 0.51 | 3.2 | 47.39 | - | 11.12 | 88.88 | 6.31 | 505.9 | 284.5 |
| RUNR | Winter 2020 | *Enteropogon* | *ramosus* | 2/07/2020 | -27.664997 | 148.69798 | 16.34 | 0.87 | 5.43 | - | - | 12.09 | 87.91 | 6.37 | 296.6 | 281.8 |
| RUNR | Winter 2020 | *Eragrostis* | *lacunaria* | 2/07/2020 | -27.662289 | 148.698881 | 17.27 | 0.78 | 4.89 | 34.79 | - | 6.05 | 93.95 | 6.74 | 330.8 | 266.3 |
| RUNR | Winter 2020 | *Lomandra* | *filiformis* | 2/07/2020 | -27.664997 | 148.69798 | 18.29 | - | - | - | - | 4.49 | 95.51 | 7.13 |  | 251.8 |
| RUNR | Winter 2020 | *Lomandra* | *filiformis* | 2/07/2020 | -27.665875 | 148.69866 | 17.92 | 1.6 | 10.03 | - | - | 3.79 | 96.21 | 6.99 | 161.3 | 256.8 |
| RUNR | Winter 2020 | *Schoenus* | *apogon* | 2/07/2020 | -27.664997 | 148.69798 | 15.04 | 1.02 | 6.36 | - | - | - | - | 5.87 | 252.9 | 305.8 |
| RUNR | Winter 2020 | *Themeda* | *triandra* | 2/07/2020 | -27.662461 | 148.698904 | 16.67 | 0.33 | 2.06 | 44.12 | - | 6.98 | 93.02 | 6.5 | 781.8 | 276.2 |
| RUNR | Winter 2020 | *Themeda* | *avenacea* | 2/07/2020 | -27.662346 | 148.697145 | 16.94 | 0.57 | 3.58 | 40.73 | - | 8.51 | 91.49 | 6.61 | 452.6 | 271.6 |
| RUNR | Winter 2020 | *Thyridolepis* | *mitchelliana* | 2/07/2020 | -27.664997 | 148.69798 | 17.21 | 1.07 | 6.69 | 37.1 | - | 7.87 | 92.13 | 6.71 | 241.1 | 267.5 |
| RUNR | Winter 2020 | *Thyridolepis* | *mitchelliana* | 2/07/2020 | -27.66574 | 148.698587 | 17.33 | 1.02 | 6.4 | - | - | - | - | 6.76 | 252.9 | 265.5 |
| RUNR | Spring 2020 | *Aristida* | *personata* | 30/09/2020 | -27.664997 | 148.69798 | 16.18 | 0.58 | 3.63 | 40.19 | - | 9.65 | 90.35 | 6.31 | 444.8 | 284.5 |
| RUNR | Spring 2020 | *Cenchrus* | *ciliaris* | 30/09/2020 | -27.664997 | 148.69798 | 15.58 | 0.63 | 3.96 | 39.07 | 66.22 | 12.49 | 87.51 | 6.08 | 409.5 | 295.2 |
| RUNR | Spring 2020 | *Chloris* | *ventricosa* | 30/09/2020 | -27.66251 | 148.69737 | 16.59 | 0.81 | 5.04 | 37.32 | - | 8.17 | 91.83 | 6.47 | 318.5 | 277.4 |
| RUNR | Spring 2020 | *Dichelachne* | *inaequiglumis* | 30/09/2020 | -27.66248 | 148.69777 | 15.77 | 0.58 | 3.65 | 38.81 | - | 10.38 | 89.62 | 6.15 | 444.8 | 291.9 |
| RUNR | Spring 2020 | *Enneapogon* | NA | 30/09/2020 | -27.66232 | 148.69902 | 16.9 | 0.69 | 4.34 | 30.41 | - | 6.52 | 93.48 | 6.59 | 373.9 | 272.4 |
| RUNR | Spring 2020 | *Enneapogon* | *nigricans* | 30/09/2020 | -27.66246 | 148.69695 | 16.02 | 0.79 | 4.96 | 33.55 | - | 8.96 | 91.04 | 6.25 | 326.6 | 287.2 |
| RUNR | Spring 2020 | *Eragrostis* | *lacunaria* | 30/09/2020 | -27.664997 | 148.69798 | 13.05 | 1.1 | 6.88 | - | - | 11.72 | 88.28 | 5.09 | 234.5 | 352.7 |
| RUNR | Spring 2020 | *Lomandra* | *filiformis* | 30/09/2020 | -27.664997 | 148.69798 | 17.45 | 0.99 | 6.16 | 37.05 | - | 3.37 | 96.63 | 6.81 | 260.6 | 263.6 |
| RUNR | Spring 2020 | *Panicum* | *effusum* | 30/09/2020 | -27.66763 | 148.70219 | 17.08 | 0.53 | 3.34 | 44.35 | 71.41 | 6.95 | 93.05 | 6.66 | 486.8 | 269.5 |
| RUNR | Spring 2020 | *Paspalidium* | *distans* | 30/09/2020 | -27.66574 | 148.698587 | 15.3 | 1.25 | 7.81 | 34.89 | - | 6.49 | 93.51 | 5.97 | 206.4 | 300.7 |
| RUNR | Spring 2020 | *Schoenus* | *apogon* | 30/09/2020 | -27.664997 | 148.69798 | 11.36 | 0.78 | 4.86 | - | - | 30.48 | 69.52 | 4.43 | 330.8 | 405.2 |
| RUNR | Spring 2020 | *Themeda* | *triandra* | 30/09/2020 | -27.66218 | 148.6988 | 16.76 | 0.34 | 2.1 | 42.4 | - | 5.91 | 94.09 | 6.54 | 758.8 | 274.5 |
| RUNR | Summer 2020/21 | *Aristida* | *latifolia* | 6/01/2021 | -27.66504 | 148.69809 | 14.14 | 0.94 | 5.85 | 30.76 | 56.37 | 26.59 | 73.41 | 5.51 | 274.5 | 325.8 |
| RUNR | Summer 2020/21 | *Cenchrus* | *ciliaris* | 6/01/2021 | -27.66504 | 148.69809 | 15.68 | 0.83 | 5.22 | 38.33 | 63.69 | 11.44 | 88.56 | 6.12 | 310.8 | 293.3 |
| RUNR | Summer 2020/21 | *Chrysopogon* | *fallax* | 6/01/2021 | -27.66504 | 148.69809 | 15.69 | 0.99 | 6.18 | 37.94 | 62.11 | 11.81 | 88.19 | 6.12 | 260.6 | 293.3 |
| RUNR | Summer 2020/21 | *Enteropogon* | *ramosus* | 6/01/2021 | -27.66504 | 148.69809 | 13.82 | 1.43 | 8.91 | 25.19 | 45.8 | 28.7 | 71.3 | 5.39 | 180.4 | 333.0 |
| RUNR | Summer 2020/21 | *Eragrostis* | *sororia* | 6/01/2021 | -27.66504 | 148.69809 | 15.07 | 1.36 | 8.52 | 23.21 | 55.2 | 20.42 | 79.58 | 5.88 | 189.7 | 305.3 |
| RUNR | Summer 2020/21 | *Lomandra* | *filiformis* | 6/01/2021 | -27.66525 | 148.69844 | 17.23 | 1.1 | 6.87 | 37.57 | 56.26 | 3.77 | 96.23 | 6.72 | 234.5 | 267.1 |
| RUNR | Summer 2020/21 | *Paspalidium* | *constrictum* | 6/01/2021 | -27.66525 | 148.69844 | 17.68 | 0.92 | 5.76 | 45.32 | 70.24 | 4.75 | 95.25 | 6.9 | 280.4 | 260.1 |
| RUNR | Summer 2020/21 | *Thyridolepis* | *mitchelliana* | 6/01/2021 | -27.66504 | 148.69809 | 15.45 | 1.35 | 8.45 | 33.03 | 55.36 | 18.39 | 81.61 | 6.03 | 191.1 | 297.7 |
| RUNR | Autumn 2021 | *Aristida* | *latifolia* | 18/04/2021 | -27.66504 | 148.69797 | 15.85 | 0.78 | 4.86 | 37.12 | 64 | 13.48 | 86.52 | 6.18 | 330.8 | 290.5 |
| RUNR | Autumn 2021 | *Aristida* | *holathera var. holathera* | 18/04/2021 | -27.66751 | 148.70222 | 15.66 | 0.61 | 3.83 | 37.06 | 67.74 | 12.56 | 87.44 | 6.11 | 423.0 | 293.8 |
| RUNR | Autumn 2021 | *Cenchrus* | *ciliaris* | 18/04/2021 | -27.66504 | 148.69797 | 15.75 | 1.08 | 6.77 | 30.68 | 62.75 | 9.89 | 90.11 | 6.14 | 238.9 | 292.3 |
| RUNR | Autumn 2021 | *Enneapogon* | *pallidus* | 18/04/2021 | -27.66661 | 148.6991 | 15.55 | 1.12 | 6.98 | 31.77 | 59.12 | 12.76 | 87.24 | 6.06 | 230.4 | 296.2 |
| RUNR | Autumn 2021 | *Enteropogon* | *acicularis* | 18/04/2021 | -27.66221 | 148.69708 | 15.07 | 1.15 | 7.18 | - | - | - | - | 5.88 | 224.3 | 305.3 |
| RUNR | Autumn 2021 | *Eragrostis* | *lacunaria* | 18/04/2021 | -27.66504 | 148.69797 | 14.12 | 0.85 | 5.32 | 28.11 | 53.79 | 19.25 | 80.75 | 5.51 | 303.5 | 325.8 |
| RUNR | Autumn 2021 | *Heteropogon* | *contortus* | 18/04/2021 | -27.66217 | 148.69854 | 15.54 | 0.51 | 3.22 | 17.74 | 48.32 | 11.27 | 88.73 | 6.06 | 505.9 | 296.2 |
| RUNR | Autumn 2021 | *Lomandra* | *filiformis* | 18/04/2021 | -27.66751 | 148.70222 | 16.96 | 1.37 | 8.53 | 42.33 | 63.16 | 4.85 | 95.15 | 6.61 | 188.3 | 271.6 |
| RUNR | Autumn 2021 | *Panicum* | *effusum* | 18/04/2021 | -27.66252 | 148.69881 | 14.69 | 0.79 | 4.91 | 30.66 | 59.16 | 16.36 | 83.64 | 5.73 | 326.6 | 313.3 |
| RUNR | Autumn 2021 | *Paspalidium* | *constrictum* | 18/04/2021 | -27.66205 | 148.69748 | 15.82 | 1.54 | 9.64 | 32.91 | 61.6 | 11.7 | 88.3 | 6.17 | 167.5 | 290.9 |
| RUNR | Autumn 2021 | *Themeda* | *avenacea* | 18/04/2021 | -27.66221 | 148.69707 | 16.04 | 0.98 | 6.1 | 38.32 | - | 7.03 | 92.97 | 6.26 | 263.3 | 286.7 |
| RUNR | Autumn 2021 | *Thyridolepis* | *mitchelliana* | 18/04/2021 | -27.66504 | 148.69797 | 15.91 | 1.32 | 8.27 | 32.78 | 59.21 | 14.33 | 85.67 | 6.2 | 195.5 | 289.5 |
| RUNR | Winter 2021 | *Aristida* | *latifolia* | 7/07/2021 | -27.66748 | 148.70247 | 15.02 | 0.6 | 3.75 | 39.28 | 64.62 | 18.03 | 81.97 | 5.86 | 430.0 | 306.3 |
| RUNR | Winter 2021 | *Cenchrus* | *ciliaris* | 7/07/2021 | -27.66498 | 148.69792 | 15.16 | 0.78 | 4.89 | 31.07 | 60.33 | 13.99 | 86.01 | 5.91 | 330.8 | 303.7 |
| RUNR | Winter 2021 | *Cymbopogon* | *refractus* | 7/07/2021 | -27.66764 | 148.7025 | 14.29 | 0.66 | 4.13 | 35.61 | 55.58 | 23.68 | 76.32 | 5.57 | 390.9 | 322.3 |
| RUNR | Winter 2021 | *Enneapogon* | *pallidus* | 7/07/2021 | -27.66498 | 148.69792 | 14.74 | 0.92 | 5.75 | 30.51 | 56.69 | 23.01 | 76.99 | 5.75 | 280.4 | 312.2 |
| RUNR | Winter 2021 | *Lomandra* | *filiformis* | 7/07/2021 | -27.66759 | 148.7027 | 16.62 | 0.92 | 5.78 | 38.81 | 58.65 | 6.28 | 93.72 | 6.48 | 280.4 | 277.0 |
| RUNR | Winter 2021 | *Panicum* | *effusum* | 7/07/2021 | -27.66261 | 148.69685 | 15.17 | 0.51 | 3.18 | 37.48 | 67.45 | 14.29 | 85.71 | 5.92 | 505.9 | 303.2 |
| RUNR | Winter 2021 | *Paspalidium* | *constrictum* | 7/07/2021 | -27.66574 | 148.69859 | 15.15 | 1.17 | 7.33 | 35.7 | 57.03 | 17.77 | 82.23 | 5.91 | 220.5 | 303.7 |
| RUNR | Winter 2021 | *Sporobolus* | *caroli* | 7/07/2021 | -27.66498 | 148.69792 | 12.5 | 0.75 | 4.69 | 25.78 | 44.49 | 32.37 | 67.63 | 4.88 | 344.0 | 367.8 |
| RUNR | Winter 2021 | *Themeda* | *triandra* | 7/07/2021 | -27.66263 | 148.69821 | 13.68 | 0.43 | 2.7 | 34.88 | 55.92 | 23.3 | 76.7 | 5.34 | 600.0 | 336.1 |
| RUNR | Winter 2021 | *Thyridolepis* | *mitchelliana* | 7/07/2021 | -27.66748 | 148.70247 | 16.16 | 0.78 | 4.9 | 37.81 | 63.67 | 11.81 | 88.19 | 6.3 | 330.8 | 284.9 |
| RUNR | Spring 2021 | *Aristida* | *latifolia* | 2/09/2021 | -27.66496 | 148.69791 | 13.66 | 0.58 | 3.64 | 32.6 | 57.17 | 25.55 | 74.45 | 5.33 | 444.8 | 336.8 |
| RUNR | Spring 2021 | *Aristida* | NA | 2/09/2021 | -27.66257 | 148.69772 | 13.79 | 0.5 | 3.13 | 35 | 58.95 | 20.34 | 79.66 | 5.38 | 516.0 | 333.6 |
| RUNR | Spring 2021 | *Austrostipa* | NA | 2/09/2021 | -27.66245 | 148.69701 | 12.5 | 0.97 | 6.06 | 26.4 | 47.25 | 32.56 | 67.44 | 4.88 | 266.0 | 367.8 |
| RUNR | Spring 2021 | *Cenchrus* | *ciliaris* | 2/09/2021 | -27.66496 | 148.69791 | 15.55 | 0.72 | 4.52 | 34.02 | 64.89 | 11.09 | 88.91 | 6.06 | 358.3 | 296.2 |
| RUNR | Spring 2021 | *Enneapogon* | *pallidus* | 2/09/2021 | -27.66235 | 148.69841 | 15.02 | 0.62 | 3.89 | 38.01 | 62.75 | 14.89 | 85.11 | 5.86 | 416.1 | 306.3 |
| RUNR | Spring 2021 | *Enteropogon* | *acicularis* | 2/09/2021 | -27.66245 | 148.69701 | 16.62 | 0.83 | 5.19 | 43.04 | 65.62 | 8.9 | 91.1 | 6.48 | 310.8 | 277.0 |
| RUNR | Spring 2021 | *Lomandra* | *filiformis* | 2/09/2021 | -27.66496 | 148.69791 | 16.11 | 1.27 | 7.95 | 35.94 | 51.84 | 11.31 | 88.69 | 6.28 | 203.1 | 285.8 |
| RUNR | Spring 2021 | *Panicum* | *effusum* | 2/09/2021 | -27.66261 | 148.69681 | 15.23 | 0.49 | 3.09 | 40.54 | 65.1 | 14.3 | 85.7 | 5.94 | 526.5 | 302.2 |
| RUNR | Spring 2021 | *Paspalidium* | *constrictum* | 2/09/2021 | -27.66529 | 148.69847 | 17.68 | 0.95 | 5.96 | 47.93 | 72.86 | 4.55 | 95.45 | 6.9 | 271.6 | 260.1 |
| RUNR | Spring 2021 | *Sporobolus* | *caroli* | 2/09/2021 | -27.66496 | 148.69791 | 14.42 | 0.93 | 5.79 | 25.78 | 52.46 | 17.65 | 82.35 | 5.62 | 277.4 | 319.4 |
| RUNR | Spring 2021 | *Thyridolepis* | *mitchelliana* | 2/09/2021 | -27.6657 | 148.69864 | 15.4 | 1.14 | 7.15 | 31.87 | 57.88 | 17.84 | 82.16 | 6.01 | 226.3 | 298.7 |

NA – Identified to Genus level only. - sample size too small to complete analysis. ^a^ Digestible energy calculated using 39% as approximation. ^b^ Daily dry matter intake (DMI) required to meet maintenance nitrogen requirement (MNR) for a 30kg wombat. ^c^ Daily dry matter intake (DMI) required to meet maintenance energy (MER) requirement for a 30kg wombat.

Table S3. Epping Forest National Park (EFNP) plant sample mineral values.

| **Site** | **Season** | **Genus** | **Species** | **Al** | **B** | **Ca** | **Co** | **Cr** | **Cu** | **Fe** | **K** | **Mg** | **Mn** | **Na** | **Ni** | **P** | **S** | **Zn** |
| --- | --- | --- | --- | --- | --- | --- | --- | --- | --- | --- | --- | --- | --- | --- | --- | --- | --- | --- |
| Units |  |  |  | mg/kg | mg/kg | % | mg/kg | mg/kg | mg/kg | mg/kg | % | % | mg/kg | % | mg/kg | % | % | mg/kg |
| Limit of Reporting | |  |  | 5.0 | 4.0 | 0.001 | 0.20 | 0.20 | 0.20 | 0.60 | 0.0004 | 0.001 | 0.10 | 0.0005 | 0.7 | 0.001 | 0.0006 | 0.80 |
| EFNP | Winter 2020 | *Heteropogon* | *contortus* | 110 | 4.80 | 0.16 | <0.40 | 0.26 | 3.70 | 84 | 0.83 | 0.08 | 94 | 0.0043 | 20 | 0.058 | 0.057 | 21 |
| EFNP | Winter 2020 | *Chrysopogon* | *fallax* | 180 | <4.0 | 0.14 | <0.40 | 0.37 | 29.00 | 150 | 0.65 | 0.07 | 58 | 0.0024 | 11 | 0.069 | 0.077 | 15 |
| EFNP | Winter 2020 | *Digitaria* | NA | 130 | <4.0 | 0.19 | <0.40 | <0.20 | 3.10 | 93 | 0.99 | 0.09 | 110 | 0.0035 | 3 | 0.090 | 0.050 | 19 |
| EFNP | Winter 2020 | *Eragrostis* | NA | 92 | <4.0 | 0.13 | <0.40 | <0.20 | 2.70 | 62 | 0.88 | 0.05 | 94 | 0.0230 | 3 | 0.080 | 0.067 | 14 |
| EFNP | Winter 2020 | *Aristida* | NA | 94 | <4.0 | 0.10 | <0.40 | <0.20 | 3.50 | 74 | 0.66 | 0.06 | 62 | 0.0060 | 2 | 0.083 | 0.076 | 27 |
| EFNP | Winter 2020 | *Triodia* | *mitchellii* | 82 | <4.0 | 0.14 | <0.40 | 0.24 | 2.70 | 81 | 0.71 | 0.05 | 240 | 0.0100 | 33 | 0.083 | 0.086 | 18 |
| EFNP | Winter 2020 | *Cenchrus* | *ciliaris* | 170 | <4.0 | 0.26 | <0.40 | 0.56 | 4.30 | 150 | 1.40 | 0.13 | 110 | 0.0082 | 19 | 0.160 | 0.072 | 20 |
| EFNP | Winter 2020 | *Themeda* | *triandra* | 100 | 5.70 | 0.17 | <0.40 | <0.20 | 3.40 | 64 | 0.67 | 0.08 | 140 | 0.0029 | 4 | 0.050 | 0.051 | 21 |
| EFNP | Winter 2020 | *Enneapogon* | NA | 130 | <4.0 | 0.17 | <0.40 | <0.20 | 2.80 | 97 | 0.44 | 0.04 | 45 | 0.0075 | 5 | 0.061 | 0.063 | 9 |
| EFNP | Winter 2020 | *Enteropogon* | *ramosus* | 170 | <4.0 | 0.16 | <0.40 | 0.26 | 3.10 | 150 | 0.37 | 0.06 | 41 | 0.0064 | 12 | 0.068 | 0.084 | 14 |
| EFNP | Winter 2020 | *Cenchrus* | *ciliaris* | 110 | <4.0 | 0.14 | <0.40 | <0.20 | 4.30 | 79 | 2.30 | 0.11 | 55 | 0.0058 | 5 | 0.097 | 0.067 | 23 |
| EFNP | Winter 2020 | *Aristida* | NA | 160 | <4.0 | 0.09 | <0.40 | <0.20 | 3.50 | 86 | 0.43 | 0.04 | 110 | 0.0029 | 8 | 0.045 | 0.061 | 13 |
| EFNP | Winter 2020 | *Fimbristylis* | *dichotoma* | 300 | 10.00 | 0.41 | <0.40 | 0.45 | 4.10 | 280 | 0.90 | 0.16 | 250 | 0.0058 | 24 | 0.130 | 0.130 | 20 |
| EFNP | Spring 2020 | *Enteropogon* | *ramosus* | 200 | <4.0 | 0.13 | <0.40 | 0.62 | 3.30 | 140 | 0.42 | 0.05 | 45 | 0.0024 | 16 | 0.096 | 0.087 | 22 |
| EFNP | Spring 2020 | *Aristida* | NA | 190 | <4.0 | 0.07 | <0.40 | 0.38 | 2.70 | 89 | 0.12 | 0.03 | 48 | 0.0024 | 15 | 0.058 | 0.050 | 12 |
| EFNP | Spring 2020 | *Enneapogon* | NA | 190 | <4.0 | 0.17 | <0.40 | 0.56 | 2.90 | 94 | 0.30 | 0.04 | 51 | 0.0056 | 5 | 0.081 | 0.063 | 12 |
| EFNP | Spring 2020 | *Cenchrus* | *ciliaris* | 510 | <4.0 | 0.29 | <0.40 | 0.98 | 3.70 | 330 | 0.57 | 0.08 | 75 | 0.0029 | 14 | 0.087 | 0.066 | 26 |
| EFNP | Spring 2020 | *Fimbristylis* | *dichotoma* | 180 | 38.00 | 0.79 | 0.47 | 0.39 | 7.30 | 190 | 4.10 | 0.36 | 230 | 0.0140 | 8 | 0.310 | 0.260 | 28 |
| EFNP | Spring 2020 | *Enneapogon* | NA | 150 | <4.0 | 0.12 | <0.40 | 0.25 | 2.20 | 110 | 0.24 | 0.04 | 69 | 0.0038 | 6 | 0.073 | 0.056 | 17 |
| EFNP | Spring 2020 | *Chrysopogon* | *fallax* | 200 | 20.00 | 0.60 | <0.40 | 0.44 | 6.60 | 190 | 2.90 | 0.28 | 180 | 0.0130 | 12 | 0.330 | 0.230 | 40 |
| EFNP | Summer 20/21 | *Chrysopogon* | *fallax* | 300 | 6.10 | 0.25 | <0.20 | 0.60 | 7.50 | 230 | 1.40 | 0.10 | 62 | 0.0037 | 14 | 0.160 | 0.160 | 20 |
| EFNP | Summer 20/21 | *Fimbristylis* | *dichotoma* | 180 | 9.90 | 0.38 | <0.20 | 0.38 | 3.70 | 160 | 1.90 | 0.17 | 530 | 0.0110 | 13 | 0.100 | 0.120 | 20 |
| EFNP | Summer 20/21 | *Aristida* | NA | 260 | 4.30 | 0.16 | <0.20 | 0.44 | 6.60 | 140 | 0.96 | 0.07 | 210 | 0.0091 | 8 | 0.100 | 0.110 | 32 |
| EFNP | Summer 20/21 | *Perotis* | *rara* | 860 | <4.0 | 0.18 | 0.68 | 1.90 | 8.80 | 670 | 1.00 | 0.10 | 210 | 0.0065 | 130 | 0.140 | 0.110 | 23 |
| EFNP | Summer 20/21 | *Enneapogon* | *robustissimus* | 170 | 4.50 | 0.20 | <0.20 | 0.31 | 4.80 | 120 | 0.80 | 0.05 | 68 | 0.0064 | 31 | 0.086 | 0.094 | 13 |
| EFNP | Summer 20/21 | *Enneapogon* | *polyphyllus* | 810 | 7.80 | 0.36 | 0.51 | 1.10 | 6.90 | 670 | 1.20 | 0.08 | 130 | 0.0054 | 47 | 0.140 | 0.130 | 22 |
| EFNP | Summer 20/21 | *Eragrostis* | NA | 480 | <4.0 | 0.12 | <0.20 | 0.85 | 8.50 | 190 | 0.76 | 0.05 | 130 | 0.0035 | 8 | 0.097 | 0.086 | 27 |
| EFNP | Summer 20/21 | *Chrysopogon* | *fallax* | 220 | 5.60 | 0.23 | <0.20 | 0.44 | 6.70 | 130 | 1.20 | 0.07 | 56 | 0.0049 | 6 | 0.120 | 0.120 | 19 |
| EFNP | Summer 20/21 | *Enteropogon* | *minutus* | 150 | <4.0 | 0.16 | <0.20 | <0.20 | 4.10 | 110 | 0.78 | 0.08 | 85 | 0.0056 | 7 | 0.120 | 0.140 | 15 |
| EFNP | Summer 20/21 | *Cenchrus* | *ciliaris* | 260 | <4.0 | 0.24 | 0.22 | 0.93 | 6.60 | 200 | 2.50 | 0.13 | 150 | 0.0140 | 10 | 0.084 | 0.110 | 38 |
| EFNP | Winter 2021 | *Eragrostis* | NA | 510 | <4.0 | 0.19 | <0.20 | 0.96 | 8.10 | 170 | 0.26 | 0.05 | 67 | 0.0072 | 6 | 0.053 | 0.061 | 18 |
| EFNP | Winter 2021 | *Heteropogon* | *contortus* | 200 | 5.40 | 0.19 | <0.20 | 0.33 | 5.90 | 100 | 0.82 | 0.10 | 150 | 0.0064 | 8 | 0.079 | 0.074 | 23 |
| EFNP | Winter 2021 | *Aristida* | NA | 170 | <4.0 | 0.17 | <0.20 | 0.29 | 3.90 | 92 | 0.17 | 0.06 | 110 | 0.0110 | 3 | 0.089 | 0.063 | 32 |
| EFNP | Winter 2021 | *Chrysopogon* | *fallax* | 350 | <4.0 | 0.21 | 0.21 | 0.45 | 5.50 | 260 | 0.36 | 0.07 | 110 | 0.0037 | 10 | 0.055 | 0.067 | 23 |
| EFNP | Winter 2021 | *Fimbristylis* | *dichotoma* | 370 | 8.70 | 0.46 | 0.56 | 0.48 | 3.10 | 250 | 0.53 | 0.11 | 620 | 0.0071 | 10 | 0.058 | 0.070 | 21 |
| EFNP | Winter 2021 | *Enneapogon* | NA | 120 | <4.0 | 0.18 | <0.20 | <0.20 | 3.10 | 78 | 0.27 | 0.04 | 62 | 0.0140 | 3 | 0.047 | 0.056 | 12 |
| EFNP | Winter 2021 | *Cenchrus* | *ciliaris* | 350 | <4.0 | 0.28 | <0.20 | 0.83 | 4.40 | 270 | 1.10 | 0.14 | 89 | 0.0077 | 10 | 0.180 | 0.077 | 30 |
| EFNP | Winter 2021 | *Triodia* | *mitchellii* | 140 | <4.0 | 0.15 | 0.24 | 0.39 | 3.50 | 100 | 0.40 | 0.04 | 360 | 0.0170 | 6 | 0.076 | 0.067 | 63 |
| EFNP | Winter 2021 | *Aristida* | NA | 230 | <4.0 | 0.14 | <0.20 | 0.33 | 4.20 | 120 | 0.17 | 0.02 | 94 | 0.0120 | 8 | 0.023 | 0.051 | 16 |

Limit of reporting - the smallest concentration that can be reported by the laboratory.

Table S4. Richard Underwood Nature Refuge (RUNR) plant sample mineral values.

| **Site** | **Season** | **Genus** | **Species** | **Al** | **B** | **Ca** | **Co** | **Cr** | **Cu** | **Fe** | **K** | **Mg** | **Mn** | **Na** | **Ni** | **P** | **S** | **Zn** |
| --- | --- | --- | --- | --- | --- | --- | --- | --- | --- | --- | --- | --- | --- | --- | --- | --- | --- | --- |
| Units |  |  |  | mg/kg | mg/kg | % | mg/kg | mg/kg | mg/kg | mg/kg | % | % | mg/kg | % | mg/kg | % | % | mg/kg |
| Limit of Reporting | |  |  | 5.0 | 4.0 | 0.001 | 0.20 | 0.20 | 0.20 | 0.60 | 0.0004 | 0.001 | 0.10 | 0.0005 | 0.7 | 0.001 | 0.0006 | 0.80 |
| RUNR | Winter 2020 | *Aristida* | NA | 550 | 22.00 | 0.21 | 1.10 | 0.83 | 7.40 | 300 | 0.51 | 0.12 | 510 | 0.0043 | 27 | 0.047 | 0.100 | 17 |
| RUNR | Winter 2020 | *Cenchrus* | *ciliaris* | 420 | 13.00 | 0.40 | <0.40 | 0.51 | 4.30 | 310 | 1.60 | 0.24 | 130 | 0.0030 | 12 | 0.220 | 0.160 | 24 |
| RUNR | Winter 2020 | *Thyridolepis* | *mitchelliana* | 450 | 4.70 | 0.13 | <0.40 | 0.48 | 4.00 | 330 | 0.42 | 0.10 | 120 | 0.0038 | 11 | 0.064 | 0.120 | 23 |
| RUNR | Winter 2020 | *Enteropogon* | *unispiceus* | 1100 | <4.0 | 0.15 | 0.55 | 1.10 | 2.50 | 730 | 0.40 | 0.05 | 59 | 0.0029 | 26 | 0.049 | 0.045 | 16 |
| RUNR | Winter 2020 | *Themeda* | *triandra* | 150 | 5.20 | 0.22 | <0.40 | <0.20 | 2.60 | 120 | 0.95 | 0.05 | 180 | 0.0056 | 12 | 0.033 | 0.040 | 16 |
| RUNR | Winter 2020 | *Themeda* | *avenacea* | 260 | 30.00 | 0.18 | <0.40 | 0.39 | 3.90 | 200 | 1.20 | 0.11 | 82 | 0.0038 | 9 | 0.048 | 0.086 | 14 |
| RUNR | Spring 2020 | *Aristida* | *personata* | 310 | 10.00 | 0.20 | <0.40 | 1.10 | 5.10 | 210 | 0.35 | 0.06 | 380 | 0.0081 | 30 | 0.060 | 0.093 | 35 |
| RUNR | Spring 2020 | *Cenchrus* | *ciliaris* | 690 | 4.30 | 0.32 | <0.40 | 1.80 | 3.80 | 450 | 0.71 | 0.14 | 110 | 0.0037 | 39 | 0.073 | 0.079 | 25 |
| RUNR | Spring 2020 | *Lomandra* | *filiformis* | 38 | 9.40 | 0.35 | <0.40 | <0.20 | 2.40 | 42 | 0.98 | 0.08 | 50 | 0.0005 | 8 | 0.069 | 0.081 | 9 |
| RUNR | Spring 2020 | *Paspalidium* | *distans* | 920 | 4.10 | 0.16 | <0.40 | 1.30 | 5.00 | 650 | 0.31 | 0.09 | 170 | 0.0055 | 62 | 0.063 | 0.140 | 34 |
| RUNR | Spring 2020 | *Panicum* | *effusum* | 590 | <4.0 | 0.18 | <0.40 | 1.00 | 3.20 | 350 | 0.19 | 0.04 | 92 | 0.0024 | 49 | 0.086 | 0.066 | 18 |
| RUNR | Spring 2020 | *Themeda* | *triandra* | 270 | 5.20 | 0.15 | <0.40 | 0.40 | 3.50 | 150 | 0.45 | 0.03 | 250 | 0.0060 | 9 | 0.036 | 0.071 | 18 |
| RUNR | Spring 2020 | *Dichelachne* | *inaequiglumis* | 510 | 5.80 | 0.18 | <0.40 | 0.76 | 3.40 | 310 | 0.18 | 0.04 | 130 | 0.0055 | 25 | 0.031 | 0.110 | 18 |
| RUNR | Spring 2020 | *Chloris* | *ventricosa* | 120 | 8.30 | 0.21 | <0.40 | 12.00 | 4.60 | 130 | 1.20 | 0.05 | 110 | 0.0065 | 5 | 0.048 | 0.180 | 11 |
| RUNR | Spring 2020 | *Enneapogon* | *nigricans* | 450 | 18.00 | 0.27 | <0.40 | 0.83 | 4.50 | 290 | 0.37 | 0.09 | 46 | 0.0045 | 14 | 0.085 | 0.100 | 23 |
| RUNR | Summer 20/21 | *Chrysopogon* | *fallax* | 630 | 6.30 | 0.24 | 0.33 | 0.69 | 3.60 | 390 | 0.61 | 0.09 | 110 | 0.0034 | 6 | 0.067 | 0.076 | 17 |
| RUNR | Summer 20/21 | *Cenchrus* | *ciliaris* | 1000 | <4.0 | 0.43 | 0.52 | 1.10 | 4.20 | 590 | 0.83 | 0.13 | 140 | 0.0037 | 5 | 0.092 | 0.071 | 18 |
| RUNR | Summer 20/21 | *Thyridolepis* | *mitchelliana* | 3700 | 4.10 | 0.15 | 1.90 | 3.50 | 7.80 | 2300 | 0.56 | 0.08 | 440 | 0.0061 | 77 | 0.092 | 0.110 | 40 |
| RUNR | Summer 20/21 | *Paspalidium* | *constrictum* | 480 | <4.0 | 0.13 | 0.38 | 0.63 | 4.40 | 280 | 0.49 | 0.04 | 120 | 0.0031 | 11 | 0.043 | 0.074 | 15 |
| RUNR | Summer 20/21 | *Lomandra* | *filiformis* | 260 | 14.00 | 0.42 | 0.21 | 0.21 | 2.90 | 160 | 0.94 | 0.12 | 42 | 0.0022 | 8 | 0.062 | 0.110 | 14 |
| RUNR | Autumn 2021 | *Panicum* | *effusum* | 4500 | <4.0 | 0.23 | 2.20 | 7.90 | 7.10 | 6100 | 1.10 | 0.12 | 130 | 0.0420 | 43 | 0.200 | 0.130 | 39 |
| RUNR | Autumn 2021 | *Thyridolepis* | *mitchelliana* | 2600 | 5.40 | 0.16 | 0.85 | 4.10 | 7.20 | 1600 | 0.82 | 0.07 | 310 | 0.0041 | 84 | 0.067 | 0.130 | 28 |
| RUNR | Autumn 2021 | *Cenchrus* | *ciliaris* | 470 | 7.30 | 0.22 | 0.24 | 0.78 | 5.90 | 370 | 2.60 | 0.13 | 130 | 0.0066 | 29 | 0.094 | 0.100 | 23 |
| RUNR | Autumn 2021 | *Aristida* | *latifolia* | 1100 | 5.90 | 0.18 | 0.49 | 2.90 | 9.00 | 720 | 0.63 | 0.06 | 530 | 0.0031 | 82 | 0.061 | 0.110 | 38 |
| RUNR | Autumn 2021 | *Paspalidium* | *constrictum* | 1500 | 5.30 | 0.36 | 0.91 | 1.80 | 6.80 | 940 | 1.60 | 0.15 | 160 | 0.0054 | 68 | 0.110 | 0.140 | 25 |
| RUNR | Winter 2021 | *Panicum* | *effusum* | 2000 | <4.0 | 0.20 | 1.00 | 2.40 | 4.80 | 1400 | 0.45 | 0.08 | 120 | 0.0130 | 74 | 0.091 | 0.078 | 33 |
| RUNR | Winter 2021 | *Aristida* | *latifolia* | 2500 | <4.0 | 0.15 | 1.10 | 5.90 | 8.00 | 1600 | 0.33 | 0.03 | 76 | 0.0022 | 220 | 0.053 | 0.065 | 22 |
| RUNR | Winter 2021 | *Cenchrus* | *ciliaris* | 1300 | 4.90 | 0.36 | 0.47 | 1.30 | 4.70 | 870 | 1.60 | 0.15 | 150 | 0.0044 | 39 | 0.065 | 0.085 | 16 |
| RUNR | Winter 2021 | *Paspalidium* | *constrictum* | 2600 | <4.0 | 0.16 | 0.86 | 8.30 | 7.50 | 1800 | 0.25 | 0.05 | 260 | 0.0020 | 230 | 0.047 | 0.099 | 21 |
| RUNR | Winter 2021 | *Lomandra* | *filiformis* | 580 | 10.00 | 1.10 | 0.34 | 0.64 | 4.00 | 390 | 1.00 | 0.12 | 53 | 0.0024 | 46 | 0.120 | 0.110 | 25 |
| RUNR | Winter 2021 | *Cymbopogon* | *refractus* | 3400 | <4.0 | 0.33 | 1.60 | 6.20 | 9.10 | 2100 | 0.48 | 0.06 | 240 | 0.0021 | 190 | 0.060 | 0.058 | 32 |
| RUNR | Spring 2021 | *Cenchrus* | *ciliaris* | 540 | 4.10 | 0.52 | 0.21 | 1.20 | 4.50 | 390 | 1.10 | 0.14 | 110 | 0.0046 | 21 | 0.095 | 0.089 | 14 |
| RUNR | Spring 2021 | *Aristida* | *latifolia* | 3000 | 7.50 | 0.16 | 0.99 | 6.60 | 9.50 | 2000 | 0.37 | 0.05 | 340 | 0.0040 | 200 | 0.038 | 0.073 | 30 |
| RUNR | Spring 2021 | *Lomandra* | *filiformis* | 1600 | 10.00 | 0.98 | 0.49 | 1.50 | 4.50 | 930 | 0.90 | 0.16 | 90 | 0.0035 | 36 | 0.076 | 0.130 | 23 |
| RUNR | Spring 2021 | *Paspalidium* | *constrictum* | 460 | <4.0 | 0.12 | 0.35 | 0.48 | 3.50 | 290 | 0.34 | 0.04 | 140 | 0.0033 | 9 | 0.036 | 0.072 | 14 |
| RUNR | Spring 2021 | *Thyridolepis* | *mitchelliana* | 2000 | <4.0 | 0.12 | 0.76 | 5.90 | 6.90 | 1400 | 0.42 | 0.05 | 250 | 0.0043 | 200 | 0.062 | 0.098 | 27 |
| RUNR | Spring 2021 | *Enneapogon* | *pallidus* | 2500 | <4.0 | 0.26 | 1.50 | 3.20 | 5.50 | 1500 | 0.23 | 0.05 | 100 | 0.0043 | 81 | 0.044 | 0.060 | 15 |
| RUNR | Spring 2021 | *Aristida* | NA | 2100 | <4.0 | 0.12 | 0.68 | 5.30 | 7.00 | 1500 | 0.18 | 0.03 | 79 | 0.0039 | 220 | 0.038 | 0.058 | 29 |
| RUNR | Spring 2021 | *Panicum* | *effusum* | 2300 | <4.0 | 0.20 | 1.20 | 2.70 | 5.30 | 1400 | 0.38 | 0.09 | 190 | 0.0120 | 43 | 0.091 | 0.088 | 49 |

Limit of reporting - the smallest concentration that can be reported by the laboratory.

Table S5. Epping Forest National Park (EFNP) scat sample nutrition values.

| **Site** | **Season** | **Collection date** | **Burrow ID** | **Latitude** | **Longitude** | **GE (MJ/kg)** | **N (%)** | | **Protein (%)** | |
| --- | --- | --- | --- | --- | --- | --- | --- | --- | --- | --- |
| EFNP | Winter 2020 | 3/06/2020 | B250 | -22.38343 | 146.70387 | 15.3718 | 1.95 | 12.19 | |  |
| EFNP | Winter 2020 | 3/06/2020 | FS1 | -22.38049 | 146.70024 | 14.3199 | 1.69 | 10.54 | |  |
| EFNP | Winter 2020 | 3/06/2020 | B275 | -22.38369 | 146.70396 | 15.5684 | 1.56 | 9.77 | |  |
| EFNP | Winter 2020 | 3/06/2020 | B61 | -22.38015 | 146.69836 | 10.2554 | 1.32 | 8.23 | |  |
| EFNP | Winter 2020 | 3/06/2020 | B104 | -22.38062 | 146.69913 | 11.9041 | 1.69 | 10.56 | |  |
| EFNP | Winter 2020 | 3/06/2020 | B66 | -22.37132 | 146.69357 | 15.4996 | 1.57 | 9.81 | |  |
| EFNP | Winter 2020 | 3/06/2020 | B48 | -22.36932 | 146.68835 | 16.6498 | 1.81 | 11.34 | |  |
| EFNP | Winter 2020 | 3/06/2020 | FS3 | -22.37201 | 146.6936 | 15.1244 | 1.64 | 10.24 | |  |
| EFNP | Winter 2020 | 3/06/2020 | B128 | -22.37072 | 146.68814 | 15.9564 | 1.52 | 9.50 | |  |
| EFNP | Spring 2020 | 3/11/2020 | B273 | -22.38394 | 146.70107 | 15.2425 | 1.43 | 8.92 | |  |
| EFNP | Spring 2020 | 3/11/2020 | B270 | -22.38346 | 146.70074 | 14.5023 | 1.44 | 9.00 | |  |
| EFNP | Spring 2020 | 3/11/2020 | B61 | -22.38023 | 146.69838 | 13.3204 | 1.61 | 10.07 | |  |
| EFNP | Spring 2020 | 3/11/2020 | B99 | -22.37824 | 146.69666 | 15.4918 | 1.82 | 11.36 | |  |
| EFNP | Spring 2020 | 3/11/2020 | B62 | -22.37974 | 146.69872 | 15.5145 | 1.50 | 9.36 | |  |
| EFNP | Spring 2020 | 3/11/2020 | B142 | -22.37877 | 146.701 | 13.3811 | 1.60 | 9.98 | |  |
| EFNP | Spring 2020 | 3/11/2020 | B96 | -22.3742 | 146.69402 | 13.9931 | 1.41 | 8.80 | |  |
| EFNP | Spring 2020 | 3/11/2020 | B96 | -22.37413 | 146.69417 | 14.3596 | 1.52 | 9.52 | |  |
| EFNP | Spring 2020 | 3/11/2020 | B140 | -22.37368 | 146.69414 | 14.1240 | 1.83 | 11.41 | |  |
| EFNP | Spring 2020 | 3/11/2020 | Adj. to B140 | -22.37354 | 146.69427 | 14.9344 | 1.75 | 10.97 | |  |
| EFNP | Spring 2020 | 3/11/2020 | B94 | -22.37314 | 146.69429 | 13.8404 | 1.26 | 7.90 | |  |
| EFNP | Spring 2020 | 3/11/2020 | B35 | -22.36 | 146.69655 | 12.8003 | 1.64 | 10.27 | |  |
| EFNP | Summer 2020/21 | 25/02/2021 | B97 | -22.37395 | 146.69327 | 12.9097 | 1.22 | 7.62 | |  |
| EFNP | Summer 2020/21 | 25/02/2021 | B71 | -22.37029 | 146.69008 | 14.1131 | 1.21 | 7.55 | |  |
| EFNP | Summer 2020/21 | 25/02/2021 | B48 | -22.36941 | 146.68842 | 12.3096 | 1.22 | 7.66 | |  |
| EFNP | Summer 2020/21 | 25/02/2021 | B70a | -22.36730 | 146.69350 | 12.9311 | 1.21 | 7.57 | |  |
| EFNP | Summer 2020/21 | 25/02/2021 | B53 | -22.36957 | 146.68916 | 13.5992 | 1.29 | 8.07 | |  |
| EFNP | Summer 2020/21 | 25/02/2021 | B65 | -22.37007 | 146.69269 | 15.0470 | 1.41 | 8.79 | |  |
| EFNP | Summer 2020/21 | 25/02/2021 | B96 | -22.37392 | 146.69431 | 13.6343 | 1.31 | 8.21 | |  |
| EFNP | Summer 2020/21 | 25/02/2021 | B70 | -22.36727 | 146.69347 | 13.3175 | 1.29 | 8.06 | |  |
| EFNP | Summer 2020/21 | 25/02/2021 | B68 | -22.36862 | 146.69228 | 10.9215 | 1.10 | 6.90 | |  |
| EFNP | Winter 2021 | 28/08/2021 | B97 | -22.37406 | 146.69330 | 14.4484 | 1.01 | 6.31 | |  |
| EFNP | Winter 2021 | 28/08/2021 | B73 | -22.37907 | 146.69746 | 14.9792 | 1.20 | 7.47 | |  |
| EFNP | Winter 2021 | 28/08/2021 | B95 | -22.37353 | 146.69353 | 15.2583 | 1.04 | 6.49 | |  |
| EFNP | Winter 2021 | 28/08/2021 | B144 | -22.37291 | 146.69337 | 11.1641 | 0.73 | 4.53 | |  |
| EFNP | Winter 2021 | 28/08/2021 | B64 | -22.37869 | 146.69678 | 15.7228 | 1.12 | 7.00 | |  |
| EFNP | Winter 2021 | 28/08/2021 | B96 | -22.37402 | 146.69424 | 16.5482 | 1.31 | 8.21 | |  |
| EFNP | Winter 2021 | 28/08/2021 | B96 | -22.37406 | 146.69414 | 16.0998 | 1.33 | 8.31 | |  |
| EFNP | Winter 2021 | 28/08/2021 | B73 | -22.37907 | 146.69746 | 16.9658 | 1.67 | 10.44 | |  |
| EFNP | Winter 2021 | 28/08/2021 | B94 | -22.37325 | 146.69432 | 12.0682 | 0.94 | 5.88 | |  |
| EFNP | Winter 2021 | 28/08/2021 | B99 | -22.37823 | 146.69172 | 16.2328 | 1.02 | 6.36 | |  |
| EFNP | Winter 2021 | 28/08/2021 | B58 | -22.37823 | 146.69172 | 15.8080 | 1.65 | 10.32 | |  |

Table S6. Richard Underwood Nature Refuge (RUNR) scat sample nutrition values.

| **Site** | **Season** | **Collection date** | **Burrow ID** | **Latitude** | **Longitude** | **GE (MJ/kg)** | **N (%)** | **Protein (%)** |
| --- | --- | --- | --- | --- | --- | --- | --- | --- |
| RUNR | Winter 2020 | 28/06/2020 | B38 | -27.668572 | 148.706115 | 16.0826 | 1.29 | 8.04 |
| RUNR | Winter 2020 | 27/06/2020 | B13 | -27.670641 | 148.707911 | 14.5254 | 1.16 | 7.26 |
| RUNR | Winter 2020 | 28/06/2020 | NA | -27.665315 | 148.70861 | 14.0758 | 1.00 | 6.26 |
| RUNR | Winter 2020 | 27/06/2020 | B36 | -27.667623 | 148.704716 | 14.3415 | 1.17 | 7.34 |
| RUNR | Winter 2020 | 28/06/2020 | WD46 | -27.66369 | 148.70854 | 15.1263 | 1.17 | 7.31 |
| RUNR | Winter 2020 | 28/06/2020 | B40 | -27.66416 | 148.702688 | 14.8342 | 1.16 | 7.24 |
| RUNR | Winter 2020 | 27/06/2020 | B31 | -27.663418 | 148.705949 | 15.1039 | 1.24 | 7.73 |
| RUNR | Winter 2020 | 28/06/2020 | B3 | -27.666153 | 148.704067 | 15.5196 | 1.28 | 7.97 |
| RUNR | Spring 2020 | 27/09/2020 | B31 | -27.663418 | 148.705949 | 10.5942 | 1.16 | 7.25 |
| RUNR | Spring 2020 | 27/09/2020 | NA | -27.662790 | 148.70605 | 11.0227 | 1.14 | 7.10 |
| RUNR | Spring 2020 | 27/09/2020 | B3 | -27.666153 | 148.704067 | 10.8008 | 1.15 | 7.22 |
| RUNR | Spring 2020 | 27/09/2020 | B30 | -27.665247 | 148.70401 | 13.9661 | 1.46 | 9.14 |
| RUNR | Spring 2020 | 28/09/2020 | B41 | -27.668821 | 148.707124 | 15.0810 | 1.63 | 10.17 |
| RUNR | Spring 2020 | 27/09/2020 | B4 | -27.665560 | 148.70506 | 13.1005 | 1.27 | 7.95 |
| RUNR | Spring 2020 | 28/09/2020 | B29 | -27.669650 | 148.70554 | 11.3972 | 1.02 | 6.35 |
| RUNR | Spring 2020 | 28/09/2020 | B27 | -27.669834 | 148.711217 | 13.7683 | 1.22 | 7.59 |
| RUNR | Summer 2020/21 | 5/01/2021 | B43 | -27.665960 | 148.707921 | 15.1732 | 1.43 | 8.95 |
| RUNR | Summer 2020/21 | 5/01/2021 | B41 | -27.668821 | 148.707120 | 12.3825 | 1.03 | 6.46 |
| RUNR | Summer 2020/21 | 6/01/2021 | B40 | -27.66416 | 148.702688 | 13.0585 | 1.31 | 8.22 |
| RUNR | Summer 2020/21 | 6/01/2021 | WD19 | -27.669609 | 148.706216 | 15.0541 | 1.32 | 8.22 |
| RUNR | Summer 2020/21 | 6/01/2021 | B29 | -27.669796 | 148.705573 | 14.4958 | 1.24 | 7.72 |
| RUNR | Summer 2020/21 | 6/01/2021 | B31 | -27.663418 | 148.705949 | 13.3402 | 1.12 | 6.99 |
| RUNR | Summer 2020/21 | 6/01/2021 | B22 | -27.662450 | 148.701432 | 14.1050 | 1.07 | 6.67 |
| RUNR | Summer 2020/21 | 6/01/2021 | B38 | -27.668572 | 148.706115 | 13.9211 | 1.17 | 7.30 |
| RUNR | Autumn 2021 | 14/04/2021 | B29 | -27.669796 | 148.705573 | 12.6117 | 1.11 | 6.94 |
| RUNR | Autumn 2021 | 15/04/2021 | B77 | -22.663690 | 148.708540 | 15.2595 | 1.40 | 8.73 |
| RUNR | Autumn 2021 | 14/04/2021 | B8 | -22.666253 | 148.708256 | 15.1999 | 1.24 | 7.78 |
| RUNR | Autumn 2021 | 14/04/2021 | NA | -22.666830 | 148.702930 | 13.5402 | 1.03 | 6.46 |
| RUNR | Autumn 2021 | 14/04/2021 | B33 | -22.669379 | 148.705230 | 14.2506 | 1.13 | 7.09 |
| RUNR | Autumn 2021 | 14/04/2021 | B33 | -22.666153 | 148.704067 | 16.0645 | 1.15 | 7.22 |
| RUNR | Autumn 2021 | 14/04/2021 | B17 | -22.669870 | 148.702844 | 12.1824 | 1.04 | 6.48 |
| RUNR | Autumn 2021 | 15/04/2021 | B27 | -22.669834 | 148.711217 | 14.4908 | 1.05 | 6.55 |
| RUNR | Winter 2021 | 7/07/2021 | B8 | -27.66253 | 148.708256 | 11.1119 | 0.88 | 5.50 |
| RUNR | Winter 2021 | 7/07/2021 | B31 | -27.663418 | 148.705949 | 16.6370 | 1.28 | 8.00 |
| RUNR | Winter 2021 | 7/07/2021 | B49 | -27.663479 | 148.698654 | 14.4719 | 1.18 | 7.37 |
| RUNR | Winter 2021 | 7/07/2021 | B28 | -27.671855 | 148.710305 | 14.6497 | 1.19 | 7.44 |
| RUNR | Winter 2021 | 7/07/2021 | B22 | -27.66245 | 148.701432 | 15.7601 | 1.21 | 7.58 |
| RUNR | Winter 2021 | 8/07/2021 | B38 | -27.668572 | 148.706115 | 15.3489 | 1.27 | 7.95 |
| RUNR | Winter 2021 | 8/07/2021 | B13 | -27.670641 | 148.707911 | 16.1409 | 1.27 | 7.96 |
| RUNR | Winter 2021 | 8/07/2021 | B12 | -27.670108 | 148.707762 | 16.2706 | 1.21 | 7.59 |
| RUNR | Spring 2021 | 2/09/2021 | B31 | -27.66341 | 148.705945 | 15.3790 | 1.28 | 7.99 |
| RUNR | Spring 2021 | 2/09/2021 | B37 | -27.66831 | 148.70379 | 15.3211 | 0.87 | 5.45 |
| RUNR | Spring 2021 | 2/09/2021 | B8 | -27.66625 | 148.70825 | 16.0343 | 1.05 | 6.55 |
| RUNR | Spring 2021 | 2/09/2021 | B6 | -27.66445 | 148.70674 | 14.4579 | 0.90 | 5.65 |
| RUNR | Spring 2021 | 2/09/2021 | B29 | -27.66979 | 148.70557 | 15.6596 | 0.87 | 5.45 |
| RUNR | Spring 2021 | 2/09/2021 | B30 | -27.66524 | 148.70471 | 16.4145 | 0.89 | 5.56 |
| RUNR | Spring 2021 | 2/09/2021 | B15 | -27.66679 | 148.70855 | 14.8361 | 0.87 | 5.46 |
| RUNR | Spring 2021 | 2/09/2021 | NA | -27.66684 | 148.70293 | 15.8213 | 0.94 | 5.86 |

Table S7. Mean faecal N (Nf) and mean plant N (Np) per sampling period.

| **Site** | **Season** | **Mean Nf** | **Mean Np** |
| --- | --- | --- | --- |
| EFNP | Winter 2020 | 1.6387 | 0.7659 |
| RUNR | Winter 2020 | 1.1828 | 0.8732 |
| EFNP | Spring 2020 | 1.5674 | 1.3219 |
| RUNR | Spring 2020 | 1.2555 | 0.7565 |
| EFNP | Summer 2020/21 | 1.2520 | 0.8719 |
| RUNR | Summer 2020/21 | 1.2105 | 1.1153 |
| RUNR | Autumn 2021 | 1.1878 | 1.0082 |
| EFNP | Winter 2021 | 1.1829 | 0.5303 |
| RUNR | Winter 2021 | 1.1829 | 0.7535 |
| RUNR | Spring 2021 | 0.9595 | 0.8201 |

Table S8. Mean faecal gross energy (GEf) and mean plant gross energy (GEp) per sampling period.

| **Site** | **Season** | **Mean GEf** | **Mean GEp** |
| --- | --- | --- | --- |
| EFNP | Winter 2020 | 14.5166 | 17.5457 |
| RUNR | Winter 2020 | 14.9511 | 16.8371 |
| EFNP | Spring 2020 | 14.2920 | 17.4908 |
| RUNR | Spring 2020 | 12.4663 | 15.6687 |
| EFNP | Summer 2020/21 | 13.1981 | 16.4272 |
| RUNR | Summer 2020/21 | 13.9413 | 15.5940 |
| RUNR | Autumn 2021 | 14.1999 | 15.5792 |
| EFNP | Winter 2021 | 15.0269 | 16.9684 |
| RUNR | Winter 2021 | 15.0489 | 14.8499 |
| RUNR | Spring 2021 | 15.4905 | 15.0893 |

Table S9. Plant genus nutritional values paired with the relative read abundance (RRA; from Casey et al. 2023) for that genus during the corresponding sampling period. Where a genus was sampled more than once in a single sampling period, values were averaged. Genera with an RRA of 0 that had been consumed during another season at that site, were included given the presumption that they were available but not eaten.

| **Site** | **Season** | **Genus** | **GE (MJ/kg)** | **Protein (%)** | **ASH (%)** | **ADF (%)** | **NDF (%)** | **RRA (%)** |
| --- | --- | --- | --- | --- | --- | --- | --- | --- |
| EFNP | Winter 2020 | *Heteropogon* | 17.1781 | 3.6184 | 5.5463 | 41.2717 | 71.0221 | 0.000694 |
| EFNP | Winter 2020 | *Chrysopogon* | 17.5615 | 5.7635 | 4.7219 | 44.2919 | 73.9882 | 0.037831 |
| EFNP | Winter 2020 | *Digitaria* | 16.8193 | 3.0883 | 7.8099 | 41.1733 | NA | 0 |
| EFNP | Winter 2020 | *Eragrostis* | 17.6664 | 2.8430 | 4.2926 | 45.7032 | NA | 0 |
| EFNP | Winter 2020 | *Aristida* | 17.7348 | 3.5022 | 3.2453 | 48.8069 | 75.8514 | 0.000513 |
| EFNP | Winter 2020 | *Cenchrus* | 17.4082 | 6.1608 | 5.6851 | 42.9984 | 73.9578 | 0.92562 |
| EFNP | Winter 2020 | *Themeda* | 17.3812 | 3.0364 | 5.5019 | 43.9273 | 71.7929 | 0 |
| EFNP | Winter 2020 | *Enneapogon* | 17.9953 | 4.2798 | 3.1224 | 46.5775 | 75.8013 | 0.002707 |
| EFNP | Winter 2020 | *Enteropogon* | 17.5587 | 3.9470 | 4.5426 | 46.7742 | 73.4895 | 7.93E-05 |
| EFNP | Winter 2020 | *Fimbristylus* | 16.7086 | 10.1906 | 8.6780 | 44.1368 | NA | 0.010127 |
| EFNP | Spring 2020 | *Enteropogon* | 18.4703 | 4.6708 | 4.0164 | 50.1162 | 77.1581 | 0 |
| EFNP | Spring 2020 | *Aristida* | 18.0112 | 2.7203 | 2.1864 | 52.3093 | 77.0182 | 0.000352 |
| EFNP | Spring 2020 | *Enneapogon* | 18.0508 | 4.2603 | 3.1111 | 49.9677 | 75.7228 | 0.000987 |
| EFNP | Spring 2020 | *Cenchrus* | 17.1531 | 5.0719 | 7.9959 | 43.4941 | 69.7254 | 0.759521 |
| EFNP | Spring 2020 | *Fimbristylus* | 16.3031 | 19.4488 | 13.1723 | 23.5095 | 43.2772 | 0.016005 |
| EFNP | Spring 2020 | *Chrysopogon* | 16.3961 | 17.4000 | 11.3886 | 27.8588 | 49.4889 | 0.178099 |
| EFNP | Summer 2020/21 | *Chrysopogon* | 16.1379 | 7.1244 | 11.2180 | 33.8962 | 66.4185 | 16.62341 |
| EFNP | Summer 2020/21 | *Fimbristylus* | 16.4085 | 6.0118 | 7.5805 | 32.8967 | 58.6572 | 5.034163 |
| EFNP | Summer 2020/21 | *Perotis* | 14.4136 | 5.7079 | 14.2738 | 32.1630 | 65.1777 | 7.731526 |
| EFNP | Summer 2020/21 | *Enneapogon* | 16.5693 | 5.3325 | 9.6145 | 42.6430 | 71.1089 | 0.622172 |
| EFNP | Summer 2020/21 | *Eragrostis* | 16.9849 | 4.2621 | 7.0926 | 43.1667 | 74.4130 | 1.890148 |
| EFNP | Summer 2020/21 | *Enteropogon* | 17.3044 | 4.3069 | 4.8558 | 47.4807 | 77.6809 | 0.003024 |
| EFNP | Summer 2020/21 | *Cenchrus* | 16.4186 | 6.3381 | 7.9655 | 36.0489 | 69.5585 | 30.80976 |
| EFNP | Winter 2021 | *Heteropogon* | 16.6585 | 2.6879 | 5.7688 | 43.9068 | 69.1069 | 0.336933 |
| EFNP | Winter 2021 | *Chrysopogon* | 16.2456 | 4.1736 | 8.3527 | 43.2275 | 69.4932 | 0.287218 |
| EFNP | Winter 2021 | *Fimbristylis* | 16.0934 | 3.8690 | 8.0655 | 45.8052 | 62.3138 | 0.551204 |
| EFNP | Winter 2021 | *Enneapogon* | 17.3296 | 3.1082 | 3.0189 | 47.2734 | 74.0234 | 0.702517 |
| EFNP | Winter 2021 | *Cenchrus* | 16.2998 | 4.6751 | 7.1399 | 35.9337 | 67.7200 | 92.49235 |
| EFNP | Winter 2021 | *Aristida* | 17.2538 | 2.4558 | 3.0145 | 51.0109 | 76.7753 | 0.231103 |
| EFNP | Winter 2021 | *Eragrostis* | 17.7456 | 2.8039 | 5.3672 | 46.2520 | 73.8964 | 0 |
| RUNR | Winter 2020 | *Cenchrus* | 15.4518 | 5.7876 | 14.9678 | 32.2743 | 60.3604 | 0.74084 |
| RUNR | Winter 2020 | *Thyridolepis* | 17.2711 | 6.5456 | 7.8747 | 37.1038 | NA | 0.020751 |
| RUNR | Winter 2020 | *Chrysopogon* | 17.4349 | 7.2825 | 6.7300 | 40.5051 | 68.5456 | 0.000227 |
| RUNR | Winter 2020 | *Eragrostis* | 17.2683 | 4.8939 | 6.0491 | 34.7900 | NA | 0.000172 |
| RUNR | Winter 2020 | *Bothriochloa* | 17.1445 | 4.1053 | 8.4611 | 40.1797 | NA | 0.000333 |
| RUNR | Winter 2020 | *Enteropogon* | 16.2658 | 4.3158 | 11.6061 | 47.3898 | NA | 0.011008 |
| RUNR | Winter 2020 | *Aristida* | 16.6165 | 5.0260 | 9.9565 | 37.9602 | NA | 0.002266 |
| RUNR | Winter 2020 | *Enneapogon* | 16.8871 | 5.7224 | 8.5905 | 48.2595 | 65.3115 | 0.000889 |
| RUNR | Summer 2020/21 | *Chrysopogon* | 15.6896 | 6.1836 | 11.8132 | 37.9352 | 62.1140 | 0.019305 |
| RUNR | Summer 2020/21 | *Cenchrus* | 15.6794 | 5.2163 | 11.4382 | 38.3254 | 63.6887 | 78.46767 |
| RUNR | Summer 2020/21 | *Thyridolepis* | 15.4495 | 8.4488 | 18.3913 | 33.0310 | 55.3570 | 0.968509 |
| RUNR | Summer 2020/21 | *aristida* | 14.1431 | 5.8478 | 26.5904 | 30.7553 | 56.3659 | 2.895865 |
| RUNR | Summer 2020/21 | *eragrostis* | 15.0654 | 8.5244 | 20.4246 | 23.2051 | 55.2042 | 0.14891 |
| RUNR | Summer 2020/21 | *enteropogon* | 13.8150 | 8.9131 | 28.6980 | 25.1919 | 45.7980 | 0.095135 |
| RUNR | Autumn 2021 | *Panicum* | 14.6893 | 4.9114 | 16.3592 | 30.6556 | 59.1635 | 1.245834 |
| RUNR | Autumn 2021 | *Thyridolepis* | 15.9081 | 8.2738 | 14.3266 | 32.7782 | 59.2141 | 8.217044 |
| RUNR | Autumn 2021 | *Cenchrus* | 15.7526 | 6.7700 | 9.8901 | 30.6800 | 62.7459 | 43.37675 |
| RUNR | Autumn 2021 | *Themeda* | 16.0394 | 6.1000 | 7.0263 | 38.3163 | NA | 0.003142 |
| RUNR | Autumn 2021 | *Aristida* | 15.7555 | 4.3459 | 13.0185 | 37.0864 | 65.8680 | 2.756157 |
| RUNR | Autumn 2021 | *enteropogon* | 15.0655 | 7.1769 | NA | NA | NA | 0.872187 |
| RUNR | Autumn 2021 | *Heteropogon* | 15.5393 | 3.2152 | 11.2658 | 17.7441 | 48.3187 | 0.201641 |
| RUNR | Autumn 2021 | *Enneapogon* | 15.5470 | 6.9825 | 12.7567 | 31.7734 | 59.1173 | 0.559551 |
| RUNR | Autumn 2021 | *Eragrostis* | 14.1173 | 5.3230 | 19.2468 | 28.1126 | 53.7921 | 1.683229 |
| RUNR | Winter 2021 | *Panicum* | 15.1730 | 3.1759 | 14.2916 | 37.4769 | 67.4511 | 0.006503 |
| RUNR | Winter 2021 | *Aristida* | 15.0199 | 3.7493 | 18.0266 | 39.2760 | 64.6160 | 0.053078 |
| RUNR | Winter 2021 | *Cenchrus* | 15.1572 | 4.8914 | 13.9946 | 31.0675 | 60.3330 | 86.14563 |
| RUNR | Winter 2021 | *Enneapogon* | 14.7426 | 5.7511 | 23.0093 | 30.5132 | 56.6893 | 0.002164 |
| RUNR | Winter 2021 | *Thyridolepis* | 16.1595 | 4.8963 | 11.8084 | 37.8085 | 63.6696 | 0.102816 |
| RUNR | Spring 2021 | *Cenchrus* | 15.5523 | 4.5246 | 11.0879 | 34.0249 | 64.8897 | 62.47146 |
| RUNR | Spring 2021 | *Sporobolus* | 14.4245 | 5.7860 | 17.6471 | 25.7793 | 52.4563 | 0.06269 |
| RUNR | Spring 2021 | *Aristida* | 13.7287 | 3.3858 | 22.9421 | 33.8016 | 58.0604 | 0.179114 |
| RUNR | Spring 2021 | *Thyridolepis* | 15.4040 | 7.1544 | 17.8423 | 31.8670 | 57.8844 | 0.310032 |
| RUNR | Spring 2021 | *Enneapogon* | 15.0162 | 3.8943 | 14.8914 | 38.0118 | 62.7469 | 0.009369 |
| RUNR | Spring 2021 | *Panicum* | 15.2260 | 3.0900 | 14.3006 | 40.5411 | 65.0951 | 0.148907 |
| RUNR | Spring 2021 | *Enteropogon* | 16.6168 | 5.1890 | 8.9034 | 43.0436 | 65.6204 | 0.056849 |
| RUNR | Spring 2021 | *Austrostipa* | 12.5004 | 6.0573 | 32.5596 | 26.4021 | 47.2489 | 0.041685 |

Figure S1: Total monthly rainfall (mm) for Epping Forest National Park (data collected on site) and Old Cashmere TM (nearest weather station to Richard Underwood Nature Refuge - RUNR) (Bureau of Meteorology, 2023). At EFNP samples were collected in June 2020, November 2020, Feb 2021, august 2021. At RUNR samples were collected in July 2020, September 2020, Jan 2021, April 2021, July 2021, September 2021.

Bureau of Meteorology, 2023. Climate Data Online. http://www.bom.gov.au/climate/data/

Accessed 10 June 2023
